# Protein target highlights in CASP15: Analysis of models by structure providers

**DOI:** 10.1002/prot.26545

**Published:** 2023-07-26

**Authors:** Leila T. Alexander, Janani Durairaj, Andriy Kryshtafovych, Luciano A. Abriata, Yusupha Bayo, Gira Bhabha, Cécile Breyton, Simon G. Caulton, James Chen, Séraphine Degroux, Damian C. Ekiert, Benedikte S. Erlandsen, Peter L. Freddolino, Dominic Gilzer, Chris Greening, Jonathan M. Grimes, Rhys Grinter, Manickam Gurusaran, Marcus D. Hartmann, Charlie J. Hitchman, Jeremy R. Keown, Ashleigh Kropp, Petri Kursula, Andrew L. Lovering, Bruno Lemaitre, Andrea Lia, Shiheng Liu, Maria Logotheti, Shuze Lu, Sigurbjörn Markússon, Mitchell D. Miller, George Minasov, Hartmut H. Niemann, Felipe Opazo, George N. Phillips, Owen R. Davies, Samuel Rommelaere, Monica Rosas‐Lemus, Pietro Roversi, Karla Satchell, Nathan Smith, Mark A. Wilson, Kuan‐Lin Wu, Xian Xia, Han Xiao, Wenhua Zhang, Z. Hong Zhou, Krzysztof Fidelis, Maya Topf, John Moult, Torsten Schwede

**Affiliations:** ^1^ Biozentrum University of Basel Basel Switzerland; ^2^ Computational Structural Biology SIB Swiss Institute of Bioinformatics Basel Switzerland; ^3^ Genome Center University of California, Davis Davis California USA; ^4^ School of Life Sciences École Polytechnique Fédérale de Lausanne Lausanne Switzerland; ^5^ Department of Biosciences University of Milano Milan Italy; ^6^ IBBA‐CNR Unit of Milano Institute of Agricultural Biology and Biotechnology Milan Italy; ^7^ Department of Cell Biology New York University School of Medicine New York New York USA; ^8^ Univ. Grenoble Alpes, CNRS, CEA, IBS Grenoble France; ^9^ The University of Birmingham Birmingham UK; ^10^ Department of Microbiology New York University School of Medicine New York New York USA; ^11^ Wellcome Centre for Cell Biology Institute of Cell Biology, University of Edinburgh Edinburgh UK; ^12^ Department of Biological Chemistry, Computational Medicine and Bioinformatics University of Michigan Ann Arbor Michigan USA; ^13^ Department of Chemistry Bielefeld University Bielefeld Germany; ^14^ Department of Microbiology, Biomedicine Discovery Institute Monash University Clayton Victoria Australia; ^15^ Securing Antarctica's Environmental Future Monash University Clayton Victoria Australia; ^16^ Centre to Impact AMR Monash University Clayton Victoria Australia; ^17^ ARC Research Hub for Carbon Utilisation and Recycling Monash University Clayton Victoria Australia; ^18^ Division of Structural Biology, Wellcome Centre for Human Genetics University of Oxford Oxford UK; ^19^ Centre for Electron Microscopy of Membrane Proteins Monash Institute of Pharmaceutical Sciences Parkville Victoria Australia; ^20^ Max Planck Institute for Biology Tübingen Germany; ^21^ Interfaculty Institute of Biochemistry, University of Tübingen Tübingen Germany; ^22^ Department of Molecular and Cell Biology, Leicester Institute of Structural and Chemical Biology University of Leicester Leicester UK; ^23^ Department of Biomedicine University of Bergen Bergen Norway; ^24^ Faculty of Biochemistry and Molecular Medicine & Biocenter Oulu University of Oulu Oulu Finland; ^25^ ISPA‐CNR Unit of Lecce Institute of Sciences of Food Production Lecce Italy; ^26^ Department of Microbiology, Immunology, and Molecular Genetics University of California Los Angeles California USA; ^27^ California NanoSystems Institute University of California Los Angeles California USA; ^28^ Lanzhou University School of Life Sciences Lanzhou China; ^29^ Department of Biosciences Rice University Houston Texas USA; ^30^ Department of Microbiology‐Immunology Northwestern Feinberg School of Medicine Chicago Illinois USA; ^31^ NanoTag Biotechnologies GmbH Göttingen Germany; ^32^ Institute of Neuro‐ and Sensory Physiology University of Göttingen Medical Center Göttingen Germany; ^33^ Center for Biostructural Imaging of Neurodegeneration (BIN) University of Göttingen Medical Center Göttingen Germany; ^34^ Department of Chemistry Rice University Houston Texas USA; ^35^ Department of Biochemistry and the Redox Biology Center University of Nebraska Lincoln Nebraska USA; ^36^ Department of Bioengineering Rice University Houston Texas USA; ^37^ University Medical Center Hamburg‐Eppendorf (UKE) Hamburg Germany; ^38^ Centre for Structural Systems Biology Leibniz‐Institut für Virologie (LIV) Hamburg Germany; ^39^ Department of Cell Biology and Molecular Genetics, Institute for Bioscience and Biotechnology Research University of Maryland Rockville Maryland USA; ^40^ Present address: Institute of Biochemistry University of Greifswald Greifswald Germany; ^41^ Present address: Department of Molecular Genetics and Microbiology University of New Mexico Albuquerque New Mexico USA

**Keywords:** CASP, cryo‐EM, protein structure prediction, X‐ray crystallography

## Abstract

We present an in‐depth analysis of selected CASP15 targets, focusing on their biological and functional significance. The authors of the structures identify and discuss key protein features and evaluate how effectively these aspects were captured in the submitted predictions. While the overall ability to predict three‐dimensional protein structures continues to impress, reproducing uncommon features not previously observed in experimental structures is still a challenge. Furthermore, instances with conformational flexibility and large multimeric complexes highlight the need for novel scoring strategies to better emphasize biologically relevant structural regions. Looking ahead, closer integration of computational and experimental techniques will play a key role in determining the next challenges to be unraveled in the field of structural molecular biology.

**Abbreviations**
ABC: ATP‐binding‐cassette
CASP: community‐wide experiment on the Critical Assessment of Techniques for Protein Structure Prediction
CNPase: 2′,3′‐cyclic nucleotide 3′‐phosphodiesterase
cryo‐EM: cryo‐electron microscopy
EDEM: ER degradation‐enhancing α‐mannosidase
ERAD: endoplasmic reticulum‐associated degradation
ICH: isocyanide hydratase
lDDT: local distance difference test
MCE: mammalian cell entry
*Mtb*
: *Mycobacterium tuberculosis*
ncAA: noncanonical amino acid
OMeY: O‐methyltyrosine
PDB: protein data bank
PDI: disulfide isomerase
RBP: receptor binding protein
RMSD: root‐mean‐square deviation
Rs: *Ralstonia solanacearum*
SAH: S‐adenosyl‐l‐homocysteine (AdoHcy)
SAM: S‐adenosyl‐l‐methionine (AdoMet)
t^6^A: N^6^‐threonylcarbamoyladenosine
TC‐AMP: threonylcarbamoyladenylate

## 1 INTRODUCTION

CASP operation would not be possible without the help of experimental structural biologists, who share their work‐in‐progress with the CASP organizing team. In the latest round of CASP (CASP15, 2022),^1^ 103 yet‐to‐be‐published structures were suggested as potential modeling targets, and 98 of them were released for prediction. They include protein–protein complexes, single‐sequence protein molecules, RNA molecules, RNA–protein complexes, and protein–ligand complexes. Five out of the 98 targets were canceled due to lack of structure at the time of evaluation, and the remaining 93 were assessed. From the assessed targets, 62 were solved by X‐ray crystallography, 27 by cryo‐EM, and 4 by NMR. The structures were provided by 48 structure determination groups from 14 countries, with the largest contribution coming from the USA (23 groups) and the UK (8). The CASP organizers, who are co‐authors of this article, thank the experimentalists who contributed to CASP15 (see Table S1) and in this way helped to develop more effective structure prediction methods for biomolecules.

This manuscript is the seventh in a series of CASP target highlight papers.^2^, ^3^, ^4^, ^5^, ^6^, ^7^ It includes reports by the authors of the selected 16 protein targets (Table S2) representing *Aquifex aeolicus* TsaB (T1183), TurandotA protein from *Drosophila melanogaster* (T1155), tyrosine O‐methyltransferase MfnG from *Streptomyces drozdowiczii* (T1124), *Mycobacterium smegmatis* Mce1 transporter (H1137), a bifunctional shikimate pathway fusion enzyme from *Clostridium* (T1180), a cryptic predatory secreted protein, Bd1399, from *B*. *bacteriovorus* (T1194), wild‐type and D180A *Ralstonia solanacearum* Isocyanide Hydratase (T1109 and T1110), bacteriophage T5 Receptor Binding Protein (RBP_pb5_) in complex with its *E*. *coli* receptor FhuA (H1129), the [NiFe]‐hydrogenase complex Huc (H1114), the ERAD misfolded glycoprotein checkpoint (H1157), the human SUN1–KASH6 complex (H1135), the myelin enzyme CNPase bound to the nanobody 8C (H1142), the nudivirus polyhedrin (T1122), a *C*. *difficile* extracellular protein of unknown function (T1176), mosquito SGS1: salivary gland surface protein 1 from *Aedes aegypti* (T1169) and type III secretion proteins YscX:YscY bound to the YscV nonamer (T1106s1, T1106s2, H1106, and H1111).

A sister paper in this issue provides reports of the RNA target providers [available online at DOI: 10.22541/au.168487314.47726735/v1]. The results of the comprehensive numerical evaluation of CASP15 models are available on the Prediction Center website (http://www.predictioncenter.org). The detailed assessment of the models by the assessors is provided elsewhere in this issue.

## 2 RESULTS

### 2.1 Structure of *Aquifex aeolicus* TsaB (CASP: T1183, PDB: 8IEY): Provided by Shuze Lu and Wenhua Zhang

N6‐threonylcarbamoyladenosine (t^6^A) is an essential post‐transcriptional modification occurring at position 37 of tRNAs that decipher ANN‐codons (N being A, U, C, or G) in all the three domains of life.^8^ The formation of tRNA t^6^A is catalyzed by two last universal common ancestor protein families of TsaC/Sua5 (COG0009)^9^ and TsaD/Kae1/Qri7 (COG0533),^10^ with support of a varying number of organism‐specific auxiliary proteins.^11^ In bacteria, tRNA t^6^A biosynthesis proceeds in two consecutive steps.^11^, ^12^, ^13^, ^14^ In the first step, TsaC utilizes *L*‐threonine, bicarbonate, and ATP to generate an intermediate threonylcarbamoyladenylate (TC‐AMP); in the second step, TsaD catalyzes the transfer of TC‐moiety from TC‐AMP onto N6 atom of tRNA A37 with support of TsaB and TsaE, leading to tRNA t^6^A. Previous studies demonstrated that TsaD, TsaB, and TsaE form an interaction network that is essential for bacterial viability.^15^ While it is hypothesized that TsaD, TsaB, and TsaE regulate the bacterial life via the tRNA t^6^A biosynthetic pathway, the molecular mechanisms of catalytic activation and cycling of these enzymes remain unknown.

Structural analyses revealed that TsaD adopts a canonical ASKHA (acetate and sugar kinase/heat shock protein 70/actin) fold with a duplicated topology βββαβαβα, which is characteristic of comprising two similar ancestral oligonucleotide‐binding domains on either side of a large cleft with an ATP‐binding site at the bottom.^11^, ^16^, ^17^, ^18^ TsaB is a paralog of TsaD^10^ and the two proteins adopt similar overall folds.^11^, ^16^, ^17^, ^18^ The N‐terminal domain of TsaB is rather conserved with the N‐terminal domain of TsaD, but the C‐terminal domain is shorter in length and does not form a nucleotide‐binding site in the cleft between the two subdomains.^11^, ^16^, ^17^, ^18^ In‐solution small‐angle X‐ray scattering (SAXS) analyses demonstrated that either TsaD or TsaB forms a homodimer, and the two readily form a heterodimer TsaD–TsaB via the same interacting interface as that in their homodimers.^11^, ^14^, ^17^ Crystal structures revealed that the interaction between TsaD and TsaB generates a conformational change in favor of binding TsaE, which forms an ATP‐mediated dynamic interaction network with TsaD–TsaB heterodimer.^14^, ^17^, ^18^

**FIGURE 1:**
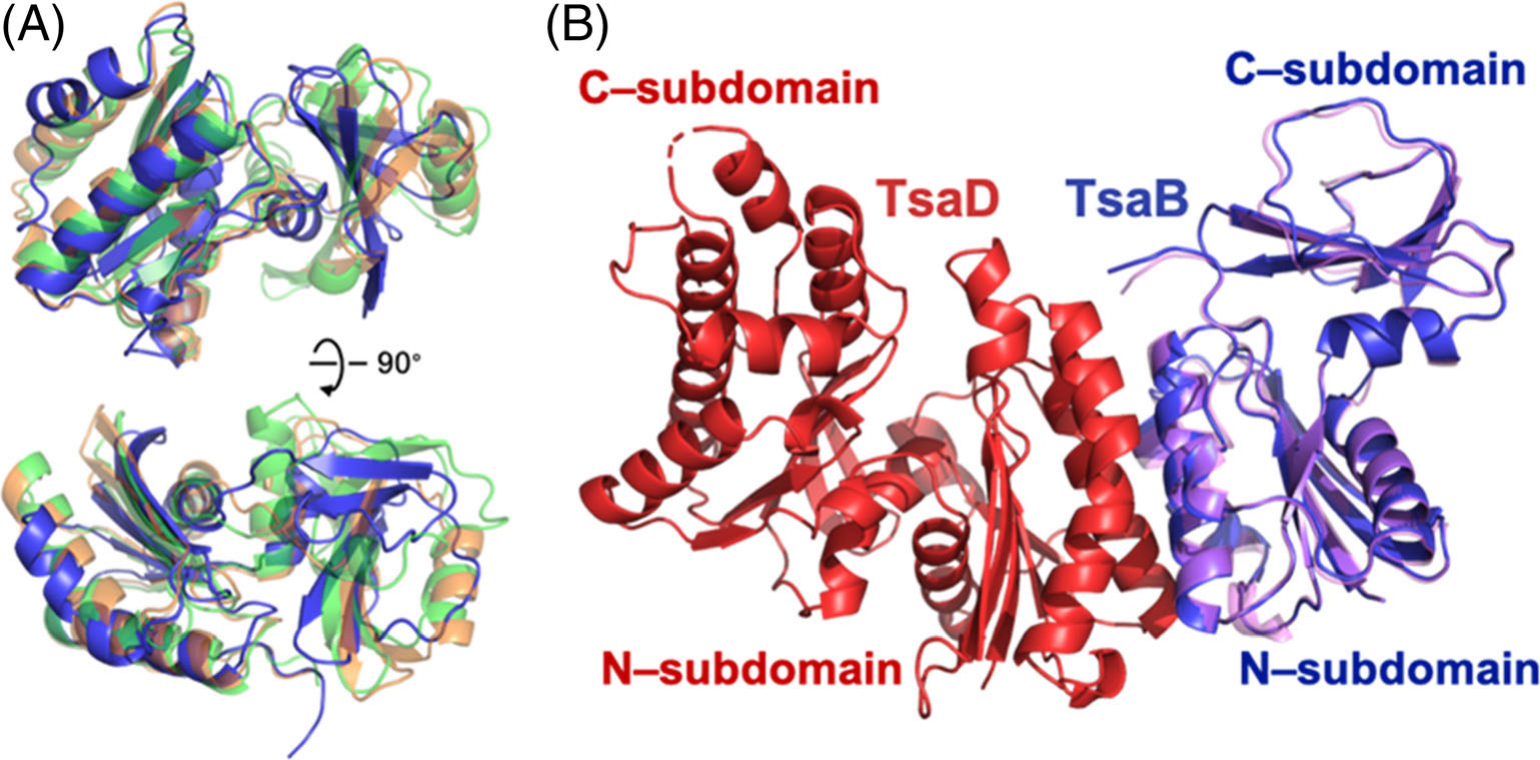
Structure of *A*. *aeolicus* TsaB. (A) The overlaid crystal structures of *E*. *coli* TsaB (PDB: 4YDU), *T*. *maritima* TsaB (PDB: 6N9A), *S*. *typhimurium* TsaB (PDB: 3ZET), *P*. *s aeruginosa* (PDB: 5BR9) and *V*. *parahaemolyticus* (PDB: 3R6M), and AlphaFold model (Entry: A0A7C5Q8I2, colored in blue). (B) Crystal structure of *A*. *aeolicus* TsaD–TsaB complex (PDB: 8IEY) with top‐scoring model (T1183TS462_1‐D1, colored in pink) of CASP 15 overlaid onto the TsaB (colored in blue).

We crystallized *A*. *aeolicus* TsaD–TsaB and collected a 2.0 Å resolution dataset for *A*. *aeolicus* TsaD–TsaB complex. The structure of TsaD was readily solved by molecular replacement using *E*. *coli* TsaD (PDB: 4YDU) as a search template. At the same time, we were unable to solve the structure of *A*. *aeolicus* TsaB using molecular replacement with available crystal structures of homologous proteins or template‐based predicted models, even though structure comparisons showed that the TsaB proteins are conserved (Figure 1A). Finally, we solved TsaB structure by molecular replacement using the predicted model (Figure 1B; entry: A0A7C5Q8I2, released on 1 November 2022) retrieved from the AlphaFold Database.^19^ The crystal structure of *A*. *aeolicus* TsaD–TsaB complex (PDB: 8IEY) revealed that TsaD interacts with TsaB via a conserved helical bundle comprising two pairs of α‐helices located in the N‐terminal regions of each protein (Figure 1B). The main structural difference resides in the C–subdomain of the TsaB proteins. For instance, Val128‐Leu136 in *A*. *aeolicus* TsaB forms a loop while all the equivalent segments in other TsaB proteins adopt an α‐helix. Remarkably, 60 highly scoring models from the CASP15 prediction results with both lDDT score better than 0.857 and the GDT‐TS better than 95.13 correctly reproduced the structure of the *A*. *aeolicus* TsaB. The top model (T1183TS462_1‐D1) predicted by MultiFOLD gave an lDDT of 0.910 and a GDT‐TS of 97.95 (Figure 1B).

### 2.2 Structure of the TurandotA protein from *Drosophila melanogaster* (CASP: T1155, PDB: N/A): Provided by Luciano A. Abriata, Samuel Rommelaere, and Bruno Lemaitre

TurandotA (TotA) belongs to a family of eight 12 kDa extracellular proteins found in *Drosophila melanogaster*.^20^ All the members of this family share a conserved sequence stretch (DGXXXQGG), called the Turandot motif.^21^ These proteins are abundantly expressed in response to a variety of stresses, including microbial infection, metabolic and osmotic stress, and temperature fluctuations; and the proteins are secreted by adipose, immune, and epithelial cells. *Turandot* gene expression is controlled by several stress and immune pathways, probably in response to tissue damage. Because of this strong and dependable transcriptional response, *Turandot* gene expression has been extensively used as a readout of *Drosophila* stress responses.^22^, ^23^ Based on their expression pattern, it was proposed that Turandots play a role in resilience to stress and may function as extracellular chaperones. However, this was never formally demonstrated, and to date, the cellular targets and molecular functions of Turandot proteins remain totally unknown. As part of our efforts to explore their possible functions, we turned to study the 3D structure of the prototypical member of the family, TotA.

**FIGURE 2:**
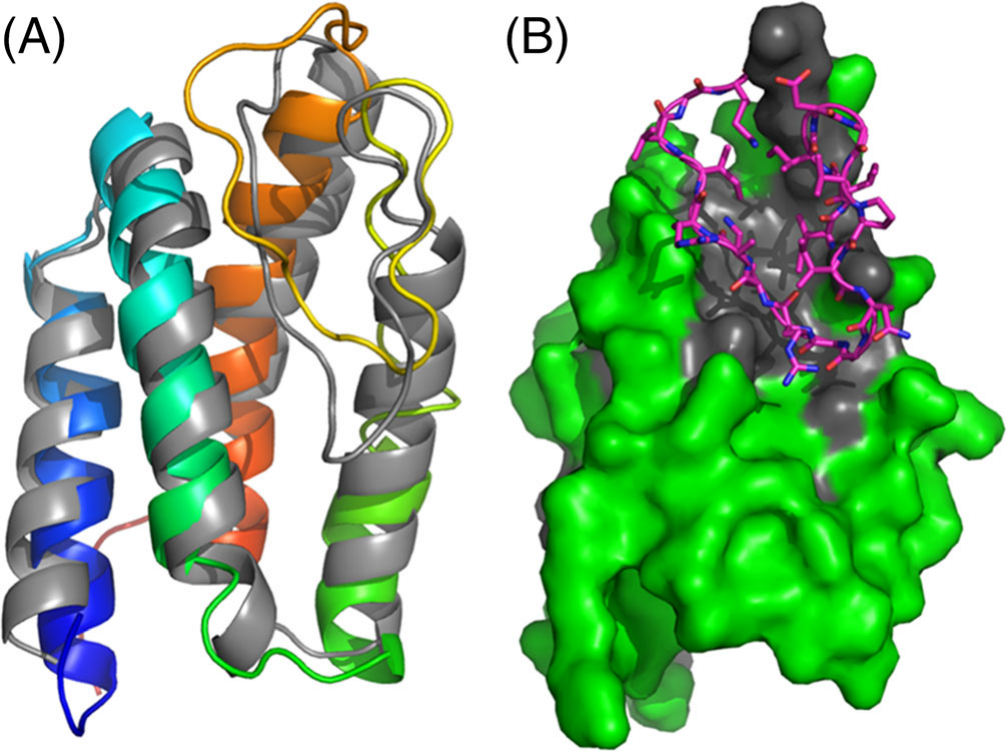
(A) Solution NMR structure of TotA, rainbow‐colored from N‐terminus (blue) to C‐terminus (red) superimposed onto the model ranked best according to GDT‐TS (gray). (B) Surface representation of the core protein colored by residue type (gray for hydrophobic amino acids, green for all others), and the flexible loop containing the Turandot motif shown as cartoons and sticks in magenta.

Recombinant TotA was impossible to crystallize due to its high solubility, but this and its small size made it perfect for NMR‐based structural characterization. With almost full assignment coverage, we solved the solution structure of TotA and characterized its dynamics through ^15^N relaxation. NMR relaxation and size‐exclusion chromatography showed that TotA is monomeric in solution (data not shown). Structure determination in solution revealed a compact core formed by four helices, with disordered termini and a poorly structured loop inserted between the third and four helices (Figure 2A). This loop is defined by fewer H–H distance restraints than the rest of the structure, and displays ^15^N relaxation parameters indicative of true dynamics in a wide range of timescales (data not shown).

The loop we identified as poorly structured and dynamic includes the Turandot motif. Despite its flexibility, the available NOEs place this loop as a lid that covers a hydrophobic surface patch at the core of the 4‐helix bundle (Figure 2B). This patch would otherwise be exposed to the solvent, which is highly unlikely for such a highly soluble protein. We are now mutating different regions of the loop in various ways to probe their effects on the biophysical properties of the protein as well as on the physiological phenotypes observed in mutant flies.

In CASP15, nearly half of the predicted TotA models, ordered by decreasing GDT‐TS, correctly capture the α‐helical core and the presence of the poorly structured loop (Figure 2A, gray). We suspect that the intrinsic nature of the loop, poorly structured, caps the GDT‐TS to 70–76 at most. In other words, the top models may actually be better than expected from the metrics. Importantly, all these top predictions place the Turandot loop and motif close to the position and conformation it adopts in our NMR structure, as a lid closing the hydrophobic patch of the helical core. Therefore, essentially, all these models would have led us to the same conclusions, and they prompt the same experiments that we devised based on the experimental structure.

### 2.3 Structure of the tyrosine O‐methyltransferase MfnG from *Streptomyces drozdowiczii* (CASP: T1124 and T1124o, PDB: 7UX8): Provided by Mitchell D. Miller, Kuan‐Lin Wu, George N. Phillips, Jr. and Han Xiao

Marformycins are anti‐infective natural products isolated from a deep‐sea sediment‐derived *Streptomyces drozdowiczii* strain. These cyclodepsipetides contain *O*‐methyl‐d‐Tyrosine. Liu et al.^24^ determined that the Tyrosine was methylated prior to incorporation by the nonribosomal peptide synthetase. They identified a SAM‐dependent O‐methyltransferase, MfnG, in the marformycins biosynthetic gene cluster and found it capable of methylating the phenoic oxygen of both d‐Tyr and l‐Tyr in vitro to produce O‐methyltyrosine (OMeY).

The properties of MfnG offered an opportunity to build a metabolic pathway for an expanded genetic code.^25^, ^26^ By autonomously biosynthesizing OMeY within an organism, we facilitate the incorporation of a noncanonical 21st amino acid in protein synthesis. We were able to selectively incorporate OMeY into proteins in *E*. *coli*, mammalian HEK293T cells, and zebrafish through genetic code expansion and metabolic engineering that included production of MfnG.^27^ This demonstrates that it is possible to generate cells and organisms that can incorporate ncAAs through exogenous biosynthesis of the ncAAs instead of high‐concentration feeding.

**FIGURE 3:**
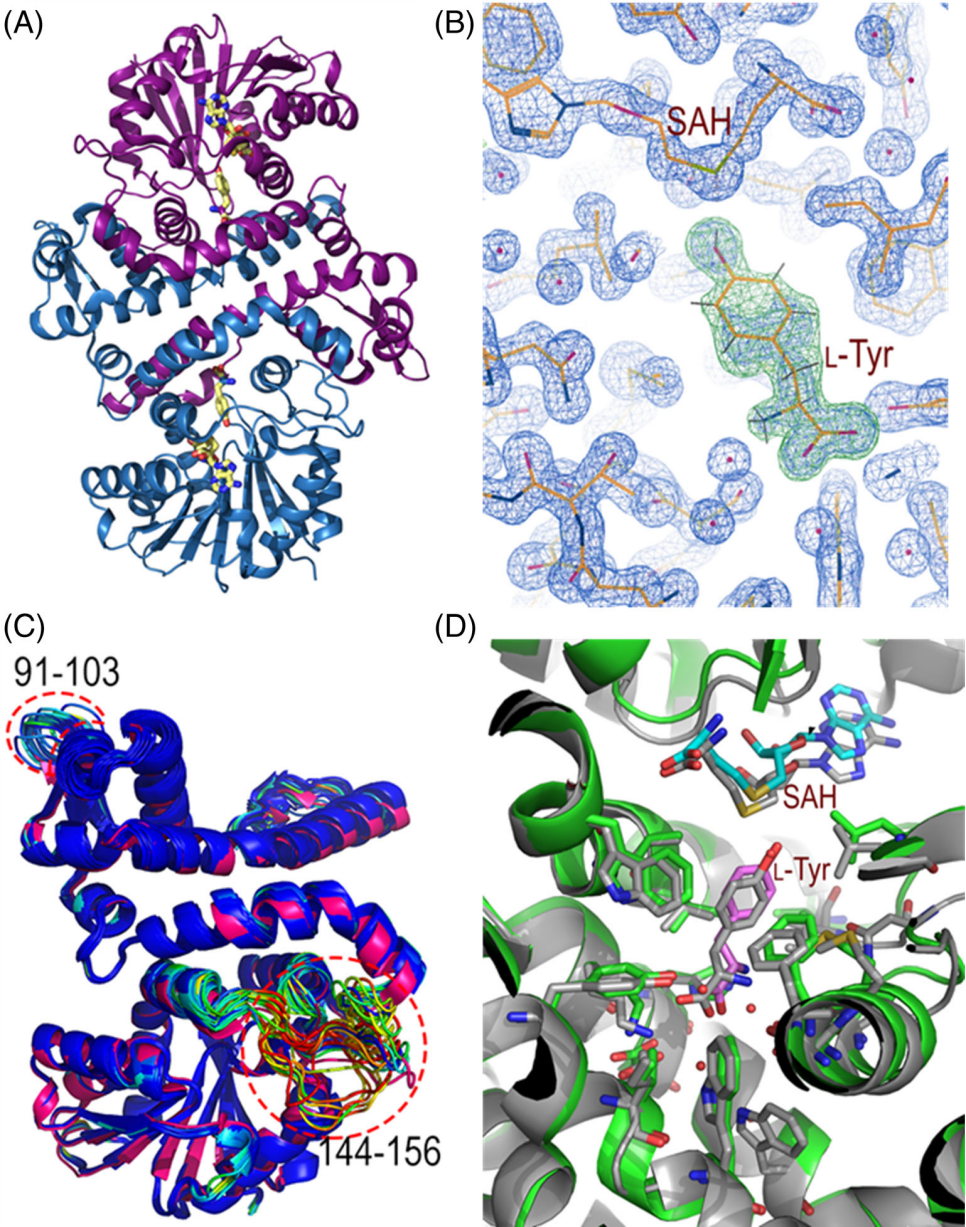
(A) View of the MfnG dimer showing the intertwining of the N‐terminal domain in dimerization along with the binding sites for the L‐Tyr and SAH. (B) Polder omits map for the Tyr (mFo‐DFc, in green contoured at +3 RMSD) with the 2mFo‐DFc omit map (in blue contoured at 1.6 RMSD) in the region of the ligands after soaking with l‐Tyr. (C) Superposition of the top quartile (rank 1–29) model 1 prediction color ramped by predicted percentage confidence estimates (with assigned scores below 60 in red and above 90 in blue) with the 7ux8 chain A structure in pink. Two residue ranges (91–103 and 144–156) that have higher B‐factors and more conformational variability across multiple crystal forms are circled. The confidence scores are lower and predictions are more varied in these regions of observed conformational variability. The N‐ (1–8) and C‐termini (364–384) are omitted for clarity. (D) Superposition of the MfnG crystal structure (7ux8, gray with water molecules near the Tyr shown in red) and the predicted structure for group 119, Kiharalab, model 1 (green), Tyr‐004 pose 2 (lilac) and SAH‐001 pose 1 (cyan). The ligand as well as the surrounding side chain atoms are in close agreement (lDDT‐PLI scores of 0.86 and 0.85 and RMSD of 0.75 and 0.89 for Tyr and SAH, respectively).

To better understand this enzyme's structural recognition and function, we determined the MfnG structure using x‐ray crystallography. Despite adding S‐adenosyl‐l‐methionine (SAM) to the protein during crystallization, we found the spent product, S‐Adenosyl‐l‐homocysteine (SAH), bound. Since the SAH is unreactive, we were able to soak in l‐Tyrosine to obtain a structure with the methyl donor product (SAH) and a methyl acceptor substrate (l‐Tyr)^27^ (Figure 3A,B).

We found MfnG could crystallize from a number of different screening conditions and that these crystals had different unit cell parameters. To date, we have phased five forms (two forms in P2_1_2_1_2_1_, two forms in P2_1_, and a P1 form), which contain 1–4 dimers (2–8 protomers) per asymmetric unit.^28^ Since the dimerization helices from one chain intertwine with the adjacent chain, they would not be expected to be seen in the same confirmation as a monomer in solution. To emphasize this, we refer to a single chain of MfnG as a protomer.

The pairwise Cα RMSD between protomers across crystal forms ranges from 0.3 to 0.5 Å. If we look at the various crystal forms, we see varying degrees of order in some of the loops, for example, 91–103 and 144–156 cannot be modeled in some forms. These are some of the same regions that have lower confidence in the predictions, suggesting that this lower confidence may reflect some mobility and flexibility in these loops (Figure 3C).

In CASP15, MfnG (T1124) was provided for monomer, homodimer, and ligand prediction categories. Given the number of other methyltransferase structures known, the protomer and homodimer predictions were classified as easy despite the nearest homolog in the PDB only having 25% sequence identity and the two protomers in the dimer being intertwined. Indeed, many of the predicted models would have been much better for molecular replacement than the homolog search model used for phasing, and these had much lower RMSDs than the experimental structure. The top quartile of model 1 predictions had lDDT between 0.85 and 0.88 and an RMSD between 1.1 and 1.8 Å for 357 matched Cα atoms, and a median RMSD of 0.8 Å for 200 residues of the C‐terminal domain, while the homolog model fragment used for phasing from the C‐terminal domain had an RMSD of 3.1 Å for 182 residues with a core of 145 residues that aligned with RMSD of 1.2 Å.

Given the conservation of the SAM/SAH binding motif within the Rossmann‐fold domain, we expected good predictions of the SAH binding poses. In fact, using AlphaFill^29^ or a similar manual method of aligning homologs from the PDB with SAH or SAM bound, one can get a reasonable starting model placing the methyl donor co‐factor within RMSD of 1.8 Å of the experimental structure despite an overall protomer Cα RMSD on the order of 2.7 Å. Many of the CASP15 predictions did much better with lDDT‐PLI scores of 0.88–0.93 and RMSDs as low as 0.37 and 0.53 Å for the two copies of SAH. The best scoring groups were Alchemy_LIG, Alchemy_LIG2, and Alchemy_LIG3, but these were often for pose number 4 or 5. Looking at only pose 1, there were several groups that ranked higher than the Alchemy groups, including ShanghaiTech, ClusPro, Kiharalab, Baker, and CoDock.

We were particularly interested to see how groups did with prediction of the l‐Tyrosine methyl acceptor. Overall, the predictions for the l‐Tyr were not as accurate as the SAH. Predicting this site is complicated by less conservation in this region. The binding of l‐Tyr involves residues from both protomers in the region of the intertwined dimerization interface and also involves some water‐mediated interactions. However, there were still several good models which had lDDT‐PLI >0.8 and ligand RMSD <1.2 Å. The closest fit was models from the Kiharalab (Figure 3C), KORP‐PL, and Grudinin groups.

In conclusion, protein–ligand complex prediction for this target proved highly successful, for both previously observed and novel poses.

### 2.4 *Mycobacterium smegmatis* Mce1 transporter (CASP: H1137, PDB: 8FEF): Provided by James Chen, Damian C. Ekiert, and Gira Bhabha

*Mycobacterium tuberculosis* (*Mtb*) is one of the leading causes of death due to infectious disease.^30^ *Mtb* infects human macrophages, where it replicates in a phagosome and scavenges nutrients from the host to survive.^31^, ^32^, ^33^, ^34^, ^35^ The Mammalian Cell Entry (MCE) protein family is involved in the import of nutrients, such as fatty acids,^36^, ^37^, ^38^, ^39^ and cholesterol,^33^, ^36^ across the cell envelope of *Mtb*. They are then utilized by the bacterium as energy sources. MCE transporters are critical virulence factors in *Mtb* and other bacterial pathogens,^33^, ^40^, ^41^, ^42^, ^43^, ^44^, ^45^ emphasizing their fundamental role in pathogenesis, but their structures and transport mechanisms are poorly understood.

**FIGURE 4:**
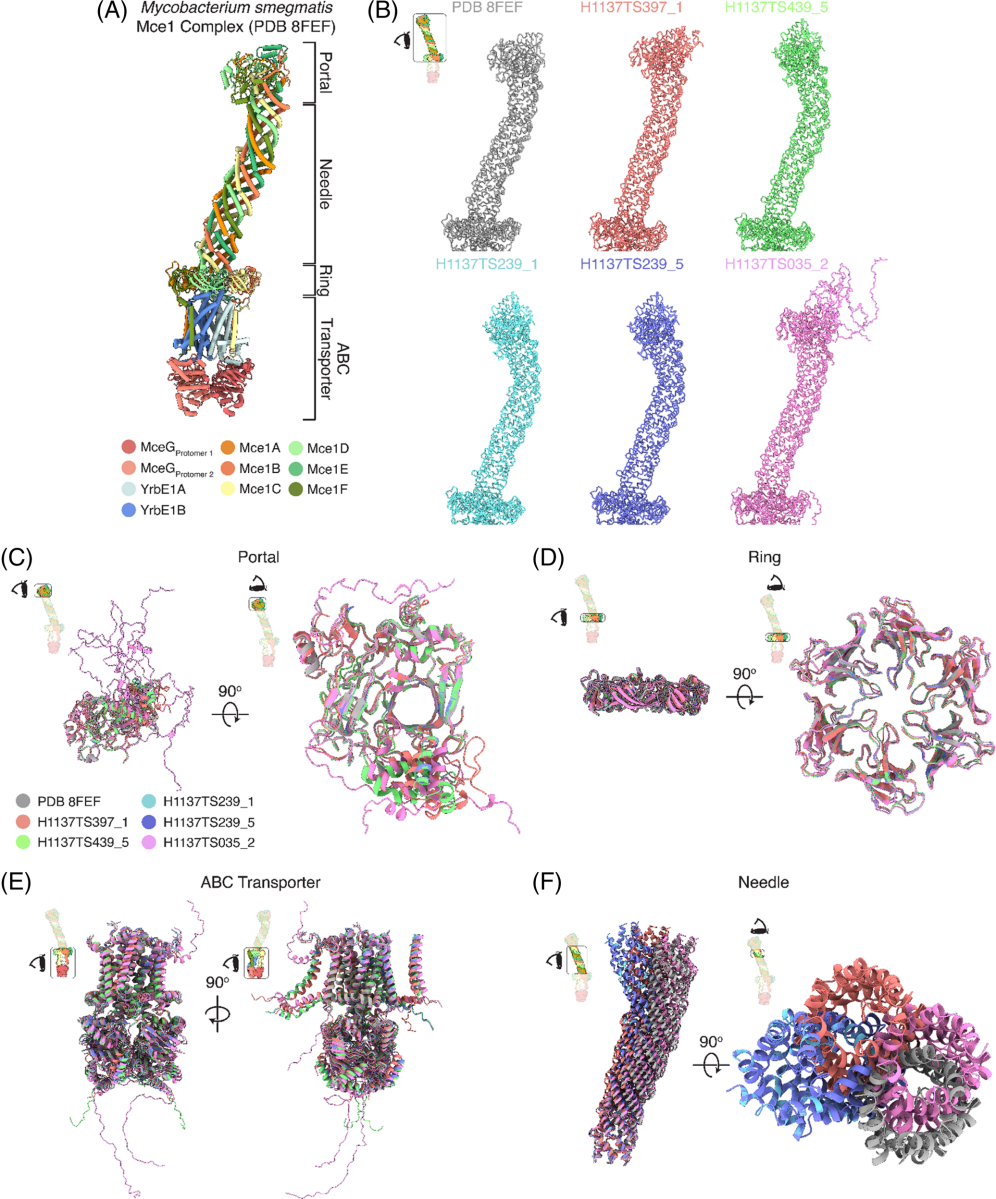
(A) Experimental cryo‐EM structure of *Mycobacterium smegmatis* Mce1 complex. Proteins are shown as cartoon cylinders and are colored by subunits according to the legend. (B) Gallery of the top five predicted structures showing the region containing the portal, needle, and ring: cryo‐EM structure (gray), H1137TS397_1 (light red), H1137TS439_5 (green), H1137TS239_1 (cyan), H1137TS239_5 (slate), and H1137TS035_2 (pink). Field of view indicated by eye‐diagram inset. (C–F) Structural alignment of the top five predicted structures with the cryo‐EM structure based on (C) portal, (D) ring, (E) ABC transporter, and (F) needle (aligned to N‐terminal end). Structures are colored according to the legend in (C). Field of view indicated by eye‐diagram inset.

Using cryo‐EM, we determined the structure of the Mce1 fatty acid transporter from *M*. *smegmatis*, a nonpathogenic relative of *Mtb*. Our structure revealed how proteins from the *mce1* operon assemble to form an unusual ATP‐binding‐cassette (ABC) transporter complex with a long hydrophobic tunnel for protected lipid transport across the bacterial cell envelope (Figure 4A).^46^ The Mce1 complex consists of 10 protein subunits: YrbE1A, YrbE1B, Mce1A, Mce1B, Mce1C, Mce1D, Mce1E, Mce1F, and two copies of MceG. Mce1 contains four major parts: (1) the portal, a globular domain at the top of the needle; (2) the needle, a curved hydrophobic tunnel created by a superhelix of 6 α‐helical segments; (3) the ring, formed by a heterohexamer of MCE domains; and (4) the ABC transporter, which consists of YrbE1AB permease and MceG ATPase subunits (Figure 4A).

*M*. *smegmatis* Mce1 (PDB: 8FEF) was provided as a multimeric modeling target in CASP15. The top five server groups generated models with QS scores between 0.890 and 0.896 that were generally in agreement with the experimental structure, with local RMSDs ranging from 4.58 to 9.10 Å. These predicted models all shared the elongated, needle‐like assembly (Figure 4B) and had the correct protein subunit stoichiometry and arrangement.

At the level of quaternary structure, the top five predicted models aligned well with the experimental structure with average α‐carbon RMSDs of 0.75 Å for the portal, 0.88 Å for the ring, 1.64 Å for the ABC transporter, and 3.34 Å for the needle. For the portal domain, the top five predictions were similar to the cryo‐EM structure, with additional predicted segments for parts of the Mce1C, Mce1D, and Mce1F C‐termini that were unresolved in the cryo‐EM map^46^ (Figure 1C). The ring domain was also well predicted in all five models with minor deviations in the loops lining the central pore (Figure 4D). Similarly, the predicted models for the ABC transporter agreed well with the cryo‐EM structure and also contained protein regions that were unresolved in the cryo‐EM map, such as the transmembrane helix of Mce1D, the N‐termini of YrbE1A and YrbE1B, and the C‐termini of the MceG homodimer (Figure 4E). However, while Mce1E is proposed to be a lipoprotein,^47^ the cleaved signal peptide was mispredicted as a transmembrane helix. Predictions of the needle domain were more variable but still generally successful (lDDT ranging from 0.797 to 0.821). The servers predicted the twisting of the α‐helical regions of Mce1ABCDEF with similar pitch and overall conformation as the cryo‐EM structure; however, the curvature of the needle varied (Figure 4F), leading to significant deviations over its ~185 Å length despite the needle appearing fairly rigid in the cryo‐EM structure.

In summary, CASP15 generated reasonably accurate models of the Mce1 complex, a 10+ subunit protein complex only distantly related to previously described protein structures. These results suggest that structure prediction methods are able to accurately predict the overall organization of some large multi‐protein complexes.

### 2.5 A bifunctional shikimate pathway fusion enzyme from *Clostridium* (CASP: T1180, PDB: N/A): Provided by Maria Logotheti and Marcus D. Hartmann

The majority of prokaryotic metabolic pathways operate via an interplay of individual monofunctional enzymes, each catalyzing distinct steps of the overall reaction cascade. While their regulation at the transcriptional level is often well understood, a potential interplay at the protein level is hard to elucidate. Although in many cases there simply may be no direct interactions, there are prominent examples of regulatory complexes between metabolic enzymes.^48^, ^49^ The question of how individual enzymes may be organized into higher‐order structures is the subject of ongoing research,^50^ with particular interest in the field of biotechnological pathway optimization.^51^

In special cases, the co‐localization of enzymes can be brought about by gene fusion events, which have been explored both by natural evolution and biotechnology.^52^ In nature, such fusions are especially prominent in eukaryotes. One such example is the pentafunctional AROM complex,^53^ which we had entered as a prediction target in CASP13.^6^ It is a large fusion enzyme conserved in the shikimate pathway in fungi and protists that attracted our attention as a long‐standing enigma: In contrast to fungi and protists, prokaryotes have the seven steps of the pathway typically encoded as individual, monofunctional enzymes. In a systematic bioinformatic analysis, we identified several exceptions to this rule, in the form of bifunctional fusion enzymes in the shikimate pathway of different prokaryotes. In the present case, we were tackling a fusion enzyme that we found in numerous species of the class Clostridia. In these, the third enzyme of the pathway, the 3‐dehydroquinate dehydratase (DHQD)^54^ is fused to the fifth enzyme, the shikimate kinase (SK).^55^ In a structural analysis, we aimed to understand if and how the two enzymes are forming a stable inter‐domain interface, which could potentially serve regulatory purposes.

**FIGURE 5:**
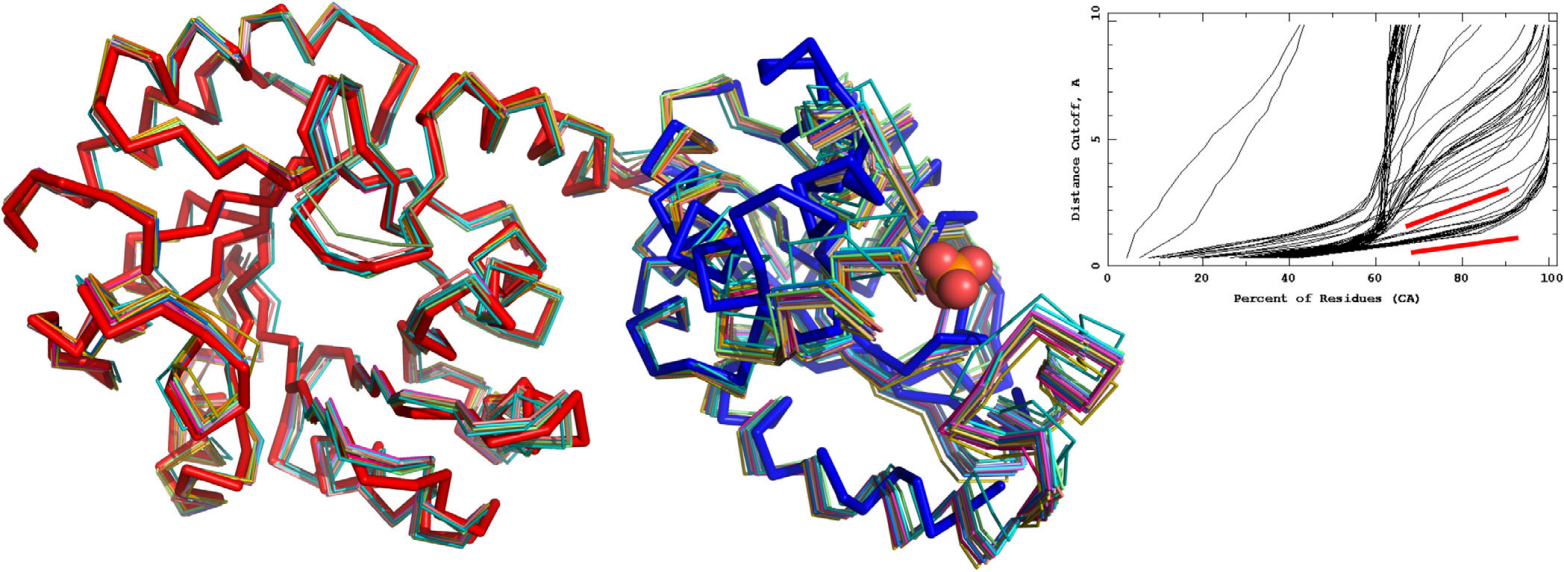
Superposition of the crystal structure of the DHQD‐SK fusion enzyme (thick ribbon, DHQD red, SK blue) and the 20 best first models (thin ribbons), which are marked in the GDT plot in the inset. The largest differences between the models and the crystal structure is found in the SK domain, which is not surprising as this domain is known to undergo larger conformational changes upon substrate binding. Nevertheless, the prediction of the inter‐domain interface closely resembles the crystal structure. As can be seen in the plot, the other models deviated often strongly in the relative positioning of the two enzymatic domains. The active site of the DHQD is at the center of the barrel, the one of the SK is marked by a phosphate molecule that co‐crystallized with the protein.

We obtained a crystal structure of this fusion enzyme from *Clostridium* sp. *CAG*:*62* (Uniprot: R7C7N8) at a resolution of 2.5 Å, showing a compact assembly (Figure 5). The structures of the two individual enzymatic domains did not bear surprises, as both the isolated (type I) DHQD, which belongs to the TIM barrel superfamily, and also the SK have been thoroughly studied in several organisms.

The focus of our analysis is the interdomain interface, with an area of 639 Å^2^ according to PISA.^56^ It is formed between the outer rim of the TIM barrel and a surface of the kinase far from its active site (Figure 5); this particular architecture appears to enable unrestricted access to both active sites from the solvent, while still forming a stable assembly. It is worth mentioning that type I DHQD typically forms homodimers. In the present fusion enzyme, however, the interface area commonly used for homodimerization is repurposed for the interface to the SK. A similar repurposing of the same interface area was previously reported in another bifunctional fusion enzyme that is conserved in plants.^57^ To the best of our knowledge, these two bifunctional fusion enzymes, the one presented here and the one found in plants, are the only known instances in which a type I DHQD is not found in its typical homodimeric form.

In CASP15, several groups did a remarkable job on this target. Obviously, the prediction of the individual domains was a trivial task, which was mastered by most groups. When it came to the prediction of the whole assembly, 20 out of the overall 92 first models submitted (i.e those which the groups marked as the best) had an overall RMSD of ≤2 Å to the crystal structure, corresponding to a GDT‐TS >83 (Figure 5). Extending the analysis to all models submitted, a total of 49 out of 433 models crossed this mark. Among 10 models with GDT‐TS > 88, eight are from the MULTICOM groups and servers, and one each from the UM‐TBM and DFolding servers. However, it cannot be excluded that the interface under examination is only forming transiently, leaving the possibility that the conformational diversity of the other predictions is reflecting a potentially dynamic situation in solution.

### 2.6 A cryptic predatory secreted protein, Bd1399, from *B*. *bacteriovorus* (CASP: T1194, PDB: 8OKH): Provided by Simon G. Caulton and Andrew L. Lovering

*B*. *bacteriovorus* is a Gram‐negative bacterium that predates other bacteria. Its characteristic lifecycle consists of prey attachment, invasion into the prey periplasm, utilization of host resources for filamentous growth, septation, prey lysis, and release of progeny.^58^ *B*. *bacteriovorus* strain HD100 encodes a large number of hypothetical proteins, and multiple large‐scale genomic and proteomic studies have attempted to identify those important for the predatory lifestyle. Lambert et al. identified 240 proteins that were upregulated by strain HD100 during predation of *Escherichia coli*,^59^ and Duncan et al. identified 104 proteins required for effective predation of *E*. *coli* by the similar 109J strain.^60^ Bd1399, comprising a putative signal peptide and DUF2846 domain, was the only protein common to both studies, highlighting its potential importance. In addition, *B*. *exovorus*, a related epibiotic predator, lacks a homolog of Bd1399, suggesting an invasion‐specific role.

**FIGURE 6:**
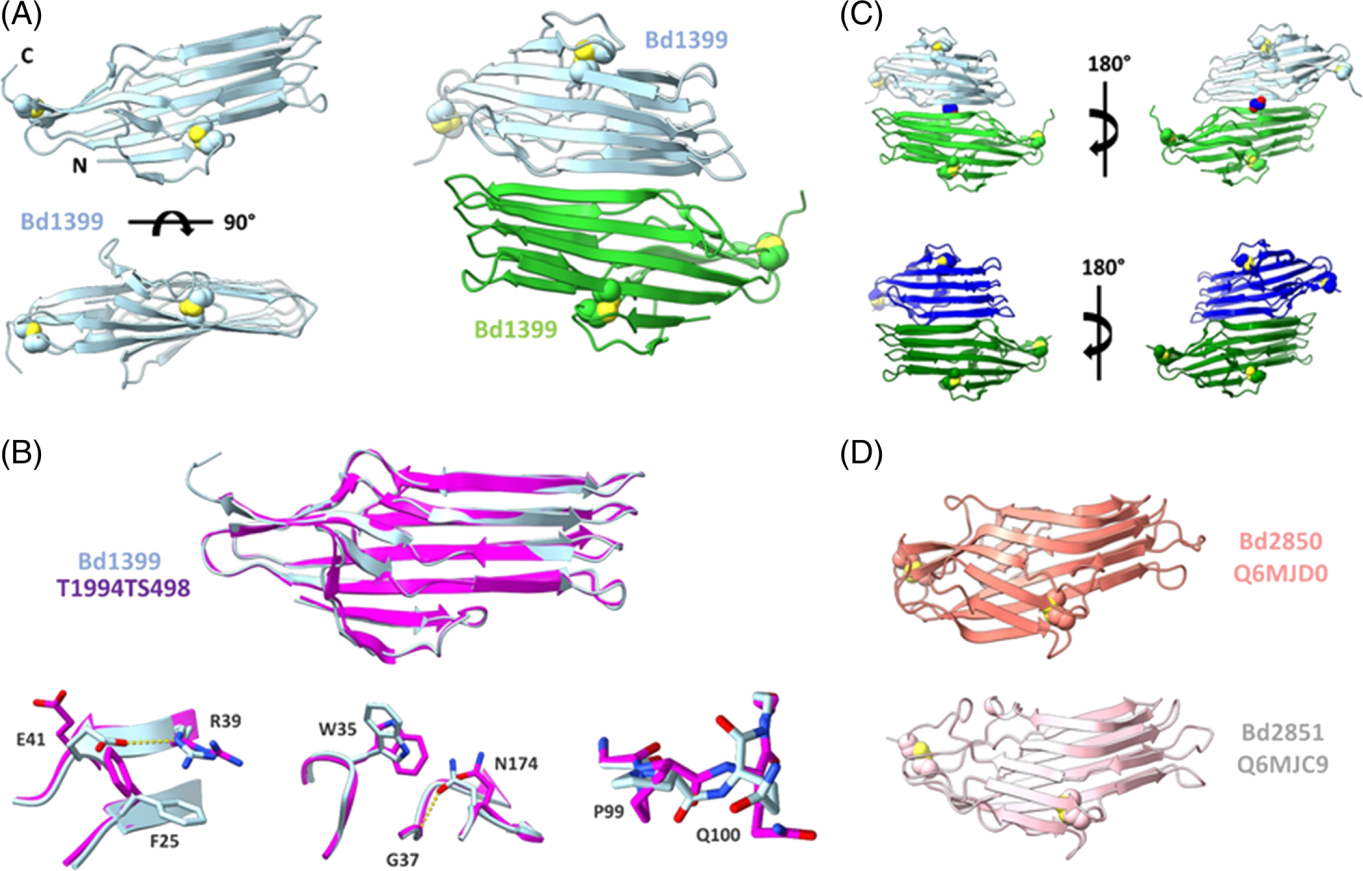
Crystal structure of Bd1399. (A) *Left*—Single chain of the Bd1399 elongated β‐sandwich with disulfides shown as spheres. Two orientations are shown 90° rotated. *Right*—The dimer observed in the asymmetric unit of the crystal. (B) *Top*—The Bd1399 dimer (light green and light blue) shows the continuous face and broken face with intercalated ethylene glycol (blue). (C) Two other *Bdellovibrio bacteriovorus* DUF4360 proteins Bd2850 and Bd2851, shown in the same orientation as Bd1399 in (A). Both proteins contain the elongated β‐sandwich and conserved disulfides observed in Bd1399. (D) *Top*—superimposed Bd1399 crystal structure (light blue) and the T1994TS498 CASP prediction (magenta). The two models show striking similarity. *Bottom*: Regions of Bd1399 that differ from the CASP prediction, show unpredicted sidechain interactions and P99‐Q100 cis‐peptide.

Purified Bd1399 was crystallized in space group P 4_3_2_1_2, and diffracted to 2.17 Å, with two molecules in the asymmetric unit. The crystal structure of Bd1399 comprises an elongated β‐sandwich that forms two relatively flat faces (Figure 6A), with a largely hydrophobic core. One face is formed from an antiparallel β‐sheet produced by strands β1, β2, β5, β8, β9, and β12; the second face exhibits a cracked antiparallel β‐sheet, comprising β3, β4, β6, β7, β10, β11, β13, and β14 (Figure 6B). A β‐bulge is formed by the residues K173 and N174, breaking the secondary structure between β10 and β11, and allowing burial of N53, Q91, and a network of water molecules into the hydrophobic core. N53 is directed inward by the preceding P52 residue, and hydrogen bonds with two buried waters and the backbone nitrogen of V175. Q91 is buried underneath strand β7, and passes under the chain between β10 and β11, hydrogen bonding with one N174‐bonded water, and the carbonyl oxygen of N174. An additional β‐bulge is caused by residues R87 and D88. The fold is locked together by two highly conserved disulfide bonds, C13–C50, which links the N‐terminal β1–β2 loop to β5, and C128–C164, which links the loop of β11–β12 to the C‐terminus.

The two molecules of Bd1399 in the asymmetric unit pack β8 around a twofold axis, producing a continuous β‐sheet across the entire dimer (Figure 6A). The opposite face does not have a continuous sheet due to intercalation of an ethylene glycol molecule between protomers (Figure 6C, top). It is unclear whether this (inferably metastable) dimer is physiological.

The structure shows Bd1399 is related to the DUF4360 family, rather than the (currently) annotated DUF2846. Searching for related structures with Foldseek^61^ identifies two additional *B*. *bacteriovorus* DUF4360 proteins, Bd2850 and Bd2851 (RMSDs of 4.05 and 4.03 Å, respectively, over 160 residues of AlphaFold^62^ models; Figure 6D). Related fungal DUF4360 family proteins are secreted by invading hyphae,^63^ and thus could imply that Bd1399 is used during the bacterial invasion process in a similar way.

The best‐performing CASP model (T1194TS498) shows remarkable similarity to that of the crystal structure, with an RMSD of 0.29 over 160 residues (168 total residues) and global lDDT of 0.82 (Figure 6B). The protein backbone of the CASP model matches the crystal structure, with few atoms displaced more than 1 Å, and well‐modeled core sidechain rotamers (Figure 6B). The largest divergence occurs in a few groups of side chains where a relay effect of one rotamer has caused a difference in a rotamer of an interacting side chain. This includes movement of the W35 and N174 sidechains, where N174 forms a H‐bond with G37 that was not observed in the crystal structure. Similarly, rotation of F25 and E41 resulted in an unobserved electrostatic interaction between E41 and R39. In addition, this model did not replicate the atypical P99‐Q100 *cis*‐peptide bond observed in the crystal structure (Figure 6D).

### 2.7 Wild‐type and D180A *Ralstonia solanacearum* isocyanide hydratase (CASP: T1109 and T1110, PDB: N/A): Provided by Nathan Smith and Mark A. Wilson

Isocyanides (also called isonitriles) are organic compounds that contain a zwitterionic triple bonded carbon–nitrogen moiety (R‐N^+^C^−^) in resonance with a double‐bonded carbenoid form, giving the isocyanide carbon atom both nucleophilic and electrophilic character. Isocyanide natural products are produced by a wide range of bacteria and fungi and possess antimicrobial^64^, ^65^ and chalkophore (copper‐binding) activities^66^, ^67^, ^68^ that confer a competitive advantage to organisms.^69^ Isocyanide hydratases (ICH) are enzymes in the DJ‐1 superfamily that use a conserved nucleophilic cysteine residue to catalyze the hydration of isocyanides to N‐formamides.^70^ ICH is thought to represent an adaptive defensive response to isocyanide natural products but may also confer protection to organisms that produce these isocyanides.

**FIGURE 7:**
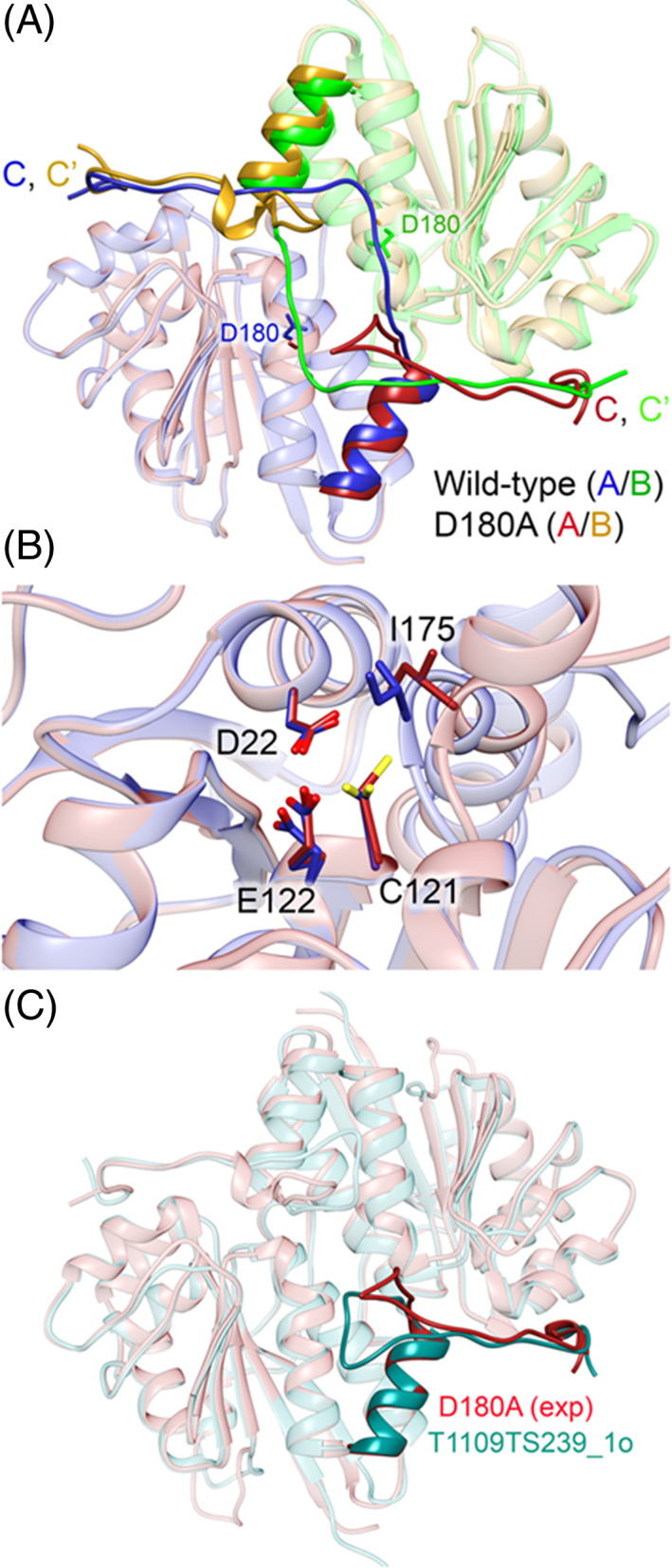
(A) The X‐ray crystal structure of the wild‐type RsICH dimer (protomer A shown in blue, and protomer B shown in green) superimposed with D180A (protomer A shown in red, and protomer B shown orange). The D180A mutation results in a reorganization of the C‐terminal region at the dimer interface (solid colors). (B) The D180A mutant (red) has different conformations of active site residues compared to the wild type (blue). The cysteine thiol of C121 faces E122 in the wild‐type structure but populates a different rotamer that faces I175 in the mutant structure. (C) Predicted model T1109TS239_1o of the D180A mutant structure (teal) reproduces the reorganization of the C‐terminal region observed in the D180A crystal structure (red).

We provided x‐ray crystal structures of wild‐type (0.74 Å resolution) and the D180A mutant (1.00 Å resolution) *Ralstonia solanacearum* ICH (RsICH) for CASP15. ICH is an obligate homodimer, and time‐resolved crystallography has shown that it forms a thioimidate intermediate with its active site cysteine nucleophile during catalysis.^71^, ^72^ The subsequent hydrolysis of this intermediate produces the N‐formamide product. Prior structural and computational analysis identified an important hydrogen bond between an aspartate (D180 in RsICH) and a tyrosine (Y204 in RsICH) that lies near the dimer interface and is involved in correlated motions that span the ICH dimer during catalysis in *Pseudomonas fluorescens* ICH.^72^ The crystal structure of the D180A RsICH mutant revealed a surprisingly large reorganization of the dimer interface and C‐terminal region, where the C‐terminus from protomer A now contacts protomer B in RsICH (Figure 7A). This is a domain swap from wild‐type RsICH, where the C‐terminus from protomer A contacts protomer A. In addition, the D180A mutation causes a change in the active site cysteine C121 rotamer (Figure 7B), likely due to structural changes near the active site caused by the C‐terminal domain swap.

Because the D180A mutation causes substantial changes in the C‐terminal region of RsICH that is intimately involved in its dimeric structure, this system provides a valuable test of current structural modeling methods' ability to predict the effects of point mutations on oligomeric protein structure. Most of the 200 top‐scoring submissions (lDDT scores 0.942–0.831) successfully predicted the wild‐type RsICH dimer and its anchoring C‐terminal disulfide at residues C147 and C220. The overall best prediction was T1110TS125_1o (lDDT score 0.935), which also had the correct active site side chain conformation of D22, C121, E122, and I175 (Figure 7B). For the more challenging case of the mutant D180A, 19 of the top 243 predictions (lDDT scores 0.899–0.796) successfully predicted the C‐terminal domain swap. The overall top prediction was T1109TS239_1o (lDDT score 0.848), which accurately predicted the C‐terminal domain swap (Figure 7C) as well as changes in C121 and I175 conformations. The C147–C220 disulfide was also correctly predicted in T1109TS239_1o, which is noteworthy because it is an inter‐protomer disulfide in D180A RsICH but an intra‐protomer bond in the wild‐type enzyme. Apart from the top 19, the other D180A RsICH models did not predict the C‐terminal rearrangement, displaying similar structures to the wild‐type enzyme. Some of these models featured a C‐terminal region that lacked the disulfide and did not make many contacts with the other portions of the protein. The prediction of structural rearrangements arising from mutations has been viewed as a significant remaining challenge in protein structure prediction,^73^ particularly for oligomeric proteins. The performance of the top CASP15 predictions of the D180A RsICH mutant structure shows that some of the newest generations of structural prediction tools can address this challenge. However, our analysis also showed that many other structure prediction methods are still biased towards experimentally determined structures with high sequence identity to the target and cannot reproduce the effects of point mutations.

### 2.8 Bacteriophage T5 receptor binding protein (RBP_pb5_ ) in complex with its *E*. *coli* receptor FhuA (CASP: H1129, PDB: 8B14): Provided by Séraphine Degroux and Cécile Breyton

Bacteriophages, phages for short, are viruses that target bacteria. The large majority of phages bear a capsid that protects the viral DNA and a tail that delivers it to the host cytoplasm. After recognizing specific host receptors via receptor binding proteins (RBPs) located at the distal end of the tail, the phage commits to infection, perforating the bacterial cell wall. Whereas many structures of RBP that recognize host saccharides are available (such as tail spike proteins or tail fibers^74^), there are still no structures of RBPs that bind protein receptors, either in apo form or bound to their receptor. In addition, the mechanism that triggers infection remains unknown. Phage T5 bears a long, flexible tail with three L‐shaped fibers at its distal end that reversibly bind a sugar moiety of the outer‐membrane lipopolysaccharides. The tail tube ends with a straight fiber, at the tip of which there is a unique protein receptor binding RBP_pb5_.^75^ The L‐fibers allow the phage to walk at the surface of the bacterium until RBP_pb5_ interacts with FhuA, an *E*. *coli* outer‐membrane transporter. Indeed, the mere interaction of RBP_pb5_ to FhuA triggers infection. We have therefore determined the structure of FhuA‐RBP_pb5_ by electron cryo‐microscopy.^76^ Comparing the structure of RBP_pb5_ within the complex with the predicted structure of RBP_pb5_ alone, together with previous biochemical and biophysical data,^77^, ^78^ we proposed a mechanism for infection trigger.^76^, ^79^ We provided the FhuA‐RBP_pb5_ complex to CASP15.

**FIGURE 8:**
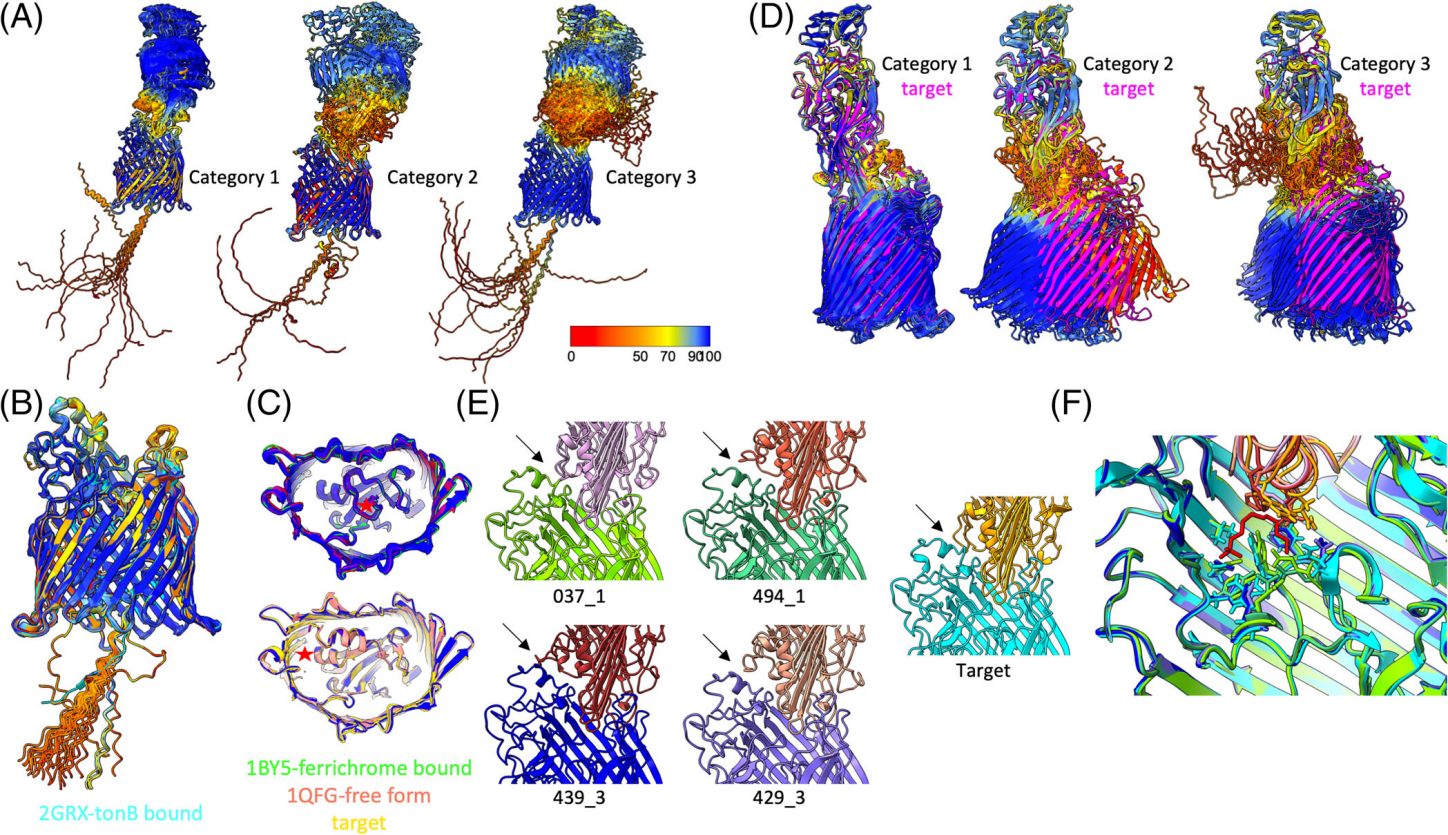
(A) Overlay on FhuA of the top 43 predictions (based on the global QS scores and the three established categories of structures) of the FhuA‐RBP_pb5_ complex, colored by prediction confidence (plDDT) and including the signal sequence. (B) Superposition of the predicted FhuA structures with the experimental FhuA structure in a complex with TonB (PDB 2GRX, cyan, TonB is not depicted). The signal peptide has been removed. (C) Periplasmic surface view of FhuA. *Top panel*: the top 42 predicted structures superimposed on Ferrichrome‐bound FhuA (PDB 1BY5, light green). *Bottom panel*: prediction 119_1/131_4 superimposed on free‐FhuA (PDB 1QFG, salmon) and FhuA from the target (PDB 8B14, yellow). *Red star*: first resolved N‐terminus of the different structures (Q18 or E19). The 1–17 residues of the predictions have been removed. (D) Superposition of the predicted structures, colored by plDDT scores, on the target RBP_pb5_ (PDB 8B14, pink), based on the three established categories of structures. (E) FhuA–RBP_pb5_ interface of the best prediction form each of the best four groups compared to that of the target. The black arrows point to areas of the interface that have fewer contacts in the predictions than in the target. (F) Superposition of the predictions presented in panel E on FhuA target (same color code as E), zoomed in on the FhuA–RBP_pb5_ interaction interface, the detergent molecule that is resolved in our structure is shown in red sticks. The residues involved in the interaction with the detergent molecule in the target are shown as sticks.

Based on the global QS and lDDT scores, we analyzed the top 43 predictions (Table 1). All groups were very confident in their predictions of FhuA β‐barrel and N‐terminal plug, and of the proximal half of RBP_pb5_ (above 90% plDDT; Figure 8A), except for the group 147 that did not propose a plDDT column, and predictions 494_1 and 165_1 that proposed average plDDT values for FhuA of RBP_pb5_ of 50% and 68%, and 30% and 82%, respectively. The sequences provided to CASP15 included FhuA's signal sequence. Only one prediction (037_1, Wallner group) proposed an α‐helix for it, the expected secondary structure when inserted in the membrane. All other predictions suggested an unstructured region with low plDDT (Figure 8A). FhuA structure has been determined with several different ligands^80^: all structures are very similar (RMSD <0.5 Å over ~675 residues for 12 structures). In all structures, the 18 first residues were not resolved, suggesting high flexibility, except when FhuA was solved in complex with TonB.^80^ All predictions proposed a random coil with low confidence for the first 18 N‐terminus residues, and none proposed the TonB‐bound fold for the TonB‐box (Figure 8B). Depending on whether a ligand is bound to FhuA, residues 18–30 adopt a different conformation. All predictions propose a ligand‐bound conformation for this sequence (Figure 8C, top panel), except for prediction 119_1 (Kiharalab group)/131_4 (Kiharalab‐Server), which proposed a ligand‐free conformation as adopted in our structure (Figure 8C, bottom panel).

**TABLE 1:** Summary table of key parameters for the 43 first predictions. Models from the same group that are in the same category are merged in the same row: models 439_1–5 include models 1, 2, 3, 4, and 5 of the Yang group. Models 133_3/434_2/011_3, 131_4/199_1, and 133_2/011_2/434_1 are identical, respectively. The column “RMSD (RBP_pb5_)” refers to the RMSD of each prediction to that of the target as determined by ChimeraX, over the 549 common residues. Other columns report figures from the CASP15 table (https://predictioncenter.org/casp15/multimer_results.cgi?target=H1129).

Category	Group	Model	QS (Global)	RMSD (RBP_pb5_), Å	lDDT (oligomer)	RMSD (interface), Å
1	Venclovas	494_1	0.852	3.253	0.822	1.69
	Wallner	037_1	0.824	3.499	0.850	1.39
	Yang	439_1‐5	0.812–0.795	3.445–3.383	0.833–0.828	1.80–2.07
	Yang‐Multimer	239_1‐5	0.773–0.705	3.494–3.348	0.827–0.815	2.00–2.58
2	CoDock	444_3‐4	0.463, 0.274	5.447, 6.786	0.721, 0.711	4.08, 6.59
	Zheng	374_2‐3	0.428, 0.384	5.418, 6.195	0.769, 0.749	6.13, 6.72
	ChaePred	398_3	0.358	6.954	0.733	5.48
	CollabFold	446_2/461_1	0.318	4.719	0.753	5.27
	FTBio0119	165_1	0.313	6.170	0.726	8.04
	ShanghaiTech	133_3/434_2/011_3	0.282	7.516	0.743	8.01
3	RaptorX	071_1	0.268	6.583	0.692	6.88
	CoDock	444_1	0.268	7.013	0.710	6.31
	McGunffin	180_1,3–5	0.266–0.214	4.062–4.016	0.780–0.770	6.35–12.40
	Kiharalab	131_4/199_1	0.265	14.846	0.711	12.07
	MUFold	298_5	0.262	12.833	0.709	8.43
	DFolding	073_3	0.244	7.855	0.695	6.79
	Zou	205_1	0.240	16.479	0.085	13.82
	SHT	147_1,3	0.237–0.234	11.824–11.733	0.669	6.92, 7.17
	ShanghaiTech	133_2/011_2/434_1	0.219	5.501	0.760	7.30
	MUFold_H	360_1,3	0.207–0.197	18.228–17.417	0.693–0.686	11.78, 15.16
	Multicom	367_4	0.197	6.657	0.727	7.51
	UltraFold	054_4	0.194	19.977	0.646	17.29

From the RBP_pb5_ perspective, predictions vary much more, and the 43 first predictions can be divided into three categories based on the global QS (QS category 1 > 0.700, 0.280 < QS category 2 < 0.275, and QS category 3 < 0.270, Figure 8D,E). All predictions proposed a correct fold for the proximal half of RBP_pb5_, consistent with their high confidence levels. This includes the N‐terminus and three long loops that are not resolved in our structure.^76^ However, predicting the distal half, which interacts with FhuA and includes seven loops, appeared to be more challenging. This could stem from the fact that these loops are predicted to be disordered in the protein alone. We proposed this disorder‐to‐order transition to be the trigger for committing the phage to infection.^76^, ^79^

The groups from the first category (494‐Venclovas, 037‐Wallner, 439‐Yang, and 239‐Yang‐Multimer) predicted both the RBP_pb5_ structure (RMSD <3.5 Å) and the FhuA–RBP_pb5_ interface (interface RMSD <2.6 Å) well. We note that in only two cases (180 and 133/011/434), the RBP_pb5_ structure was well predicted but the FhuA–RBP_pb5_ interface was not (Figure 8E). In the best cases, the prediction of the interface is close to the target, however with fewer interactions (arrow in Figure 8F). Interestingly, in our structure, we resolved a detergent molecule at the interface, which could not be replicated in the predictions as this information was not available. However, the network of residues stabilizing the detergent molecule is quite well predicted (Figure 8G).

To conclude, despite the lack of experimentally determined receptor RBP structures, several groups successfully reproduced the RBP_pb5_ structure and to a certain extent the FhuA‐RBP_pb5_ interface. Exceptionally, one group reproduced the solved conformation of FhuA, however, no group was able to correctly predict the entire complex with high accuracy.

### 2.9 The structure of the [NiFe]‐hydrogenase complex Huc (CASP: H1114, PDB: 7UUS, 7UTD, 7UUR, 8DQV): Provided by Rhys Grinter, Ashleigh Kropp, and Chris Greening

Huc is a member of the widespread [NiFe]‐hydrogenase family^81^, ^82^, ^83^ of enzymes that catalyze the interconversion of molecular hydrogen (H_2_) into two protons and two electrons.^84^ Huc is utilized by the bacterium *Mycobacterium smegmatis* to convert the trace quantities of H_2_ in the air into energy to support growth and maintenance when other energy sources are limited.^85^ As such, this enzyme has extremely high affinity to hydrogen and can catalyze the oxidation of oxygen even at concentrations well below atmospheric.^83^ Additionally, while most other hydrogenases are strongly inhibited by molecular oxygen (O_2_), Huc is insensitive to it, which is an important prerequisite to oxidizing hydrogen in air.^83^, ^86^

**FIGURE 9:**
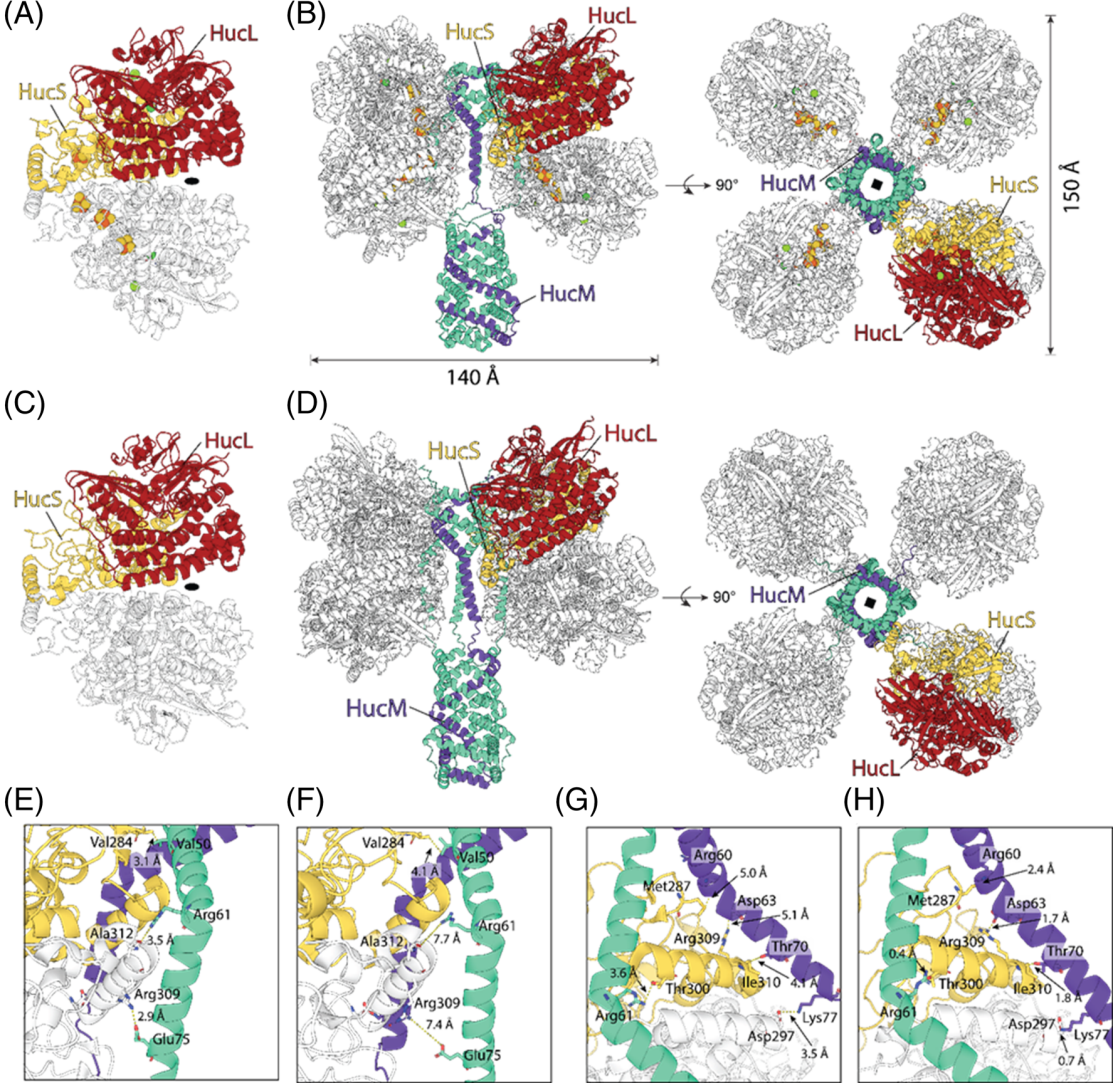
Comparison of the experimental structure of Huc with the best‐scored CASP15 model. (A) The cryoEM structure of a HucS_2_L_2_ lobe from the Huc complex. One HucS subunit is colored yellow, and one HucL subunit is colored red. [3Fe4S] clusters are shown as yellow and orange spheres, the Ni ion from the NiFe active site is shown as a green sphere, and an Mg ion is shown as a lime sphere. (B) The cryoEM structure of the Huc complex. One HucSL dimer and co‐factors are colored as in panel a. One HucM molecule is colored blue, and the others are colored green. (C) Yang group computational model 1 (H1114TS439_1) of a HucS_2_L_2_ lobe, colored as in panel A. (D) The H1114TS439_1 model of the Huc complex, colored as in panel B. (E and G) Zoomed views of the HucS_2_L_2_‐HucM interface of the cryoEM structure, and (F and H) the H1114TS439_1 model of Huc.

Huc forms an 833 kDa complex composed of three protein subunits, HucL, HucS, and HucM. The Huc complex is composed of 8 HucL, 8 HucS, and 4 HucM molecules. The HucL and HucS subunits are the canonical components of an [NiFe]‐hydrogenase, constituting the catalytic and electron‐transferring subunits of these enzymes respectively, and forming a heterodimer. In the Huc complex, the HucSL catalytic promoters further dimerize to form four heart‐shaped lobes (named HucS_2_L_2_), each of which contains interconnected electron transfer relays (Figure 9A). HucM has an elongated helical structure, with the four subunits present in the complex forming an intertwined tetramer that acts as a scaffold for the four HucS_2_L_2_ lobes around a C4 symmetry axis (Figure 9B). The HucM tetramer also mediates a peripheral association with the inner face of the cytoplasmic membrane via a hollow helical tube lined with hydrophobic residues that allow menaquinone, the electron acceptor for Huc, to enter a hydrophobic chamber at the centre of the complex.^83^ Menaquinone binds to the electron acceptor substrate binding sites in the complex and is reduced to menaquinol by electrons from the oxidation of atmospheric hydrogen. Reduced menaquinol then diffuses back into the membrane, where it is oxidized by a terminal oxidase to generate proton motive force for the cells.^81^, ^83^

While CASP14 and the subsequent release of the AlphaFold2 code demonstrated that machine learning‐based approaches are highly successful at modeling protein structure,^7^, ^62^, ^87^ we felt that the size and complexity of the Huc complex would represent a considerable challenge to structural modeling software. As such, we thought it was an excellent target for CASP15 to test the new developments in protein structure prediction. We were not disappointed as a number of teams did an impressive job of modeling the Huc complex. Teams including Yang (G439), Zheng (G374), Venclovas (G494), Kiharalab (G119), Manifold (G248), and McGuffin (G180) accurately reproduced the overall architecture of Huc in at least some of their models. The size and flexibility of the Huc complex made it difficult to assess the quality of these models using a single metric (e.g., lDDT, QS, TM‐score). TM‐score appeared to be the best indicator of model quality from our perspective, while some models with high lDDT scores were incorrect (e.g., Ultrafold (G054) model 4 (H1114TS054_4) with lDDT score of 0.866), and QS scores alone were a poor estimator of the accuracy of the Huc complex models. This is a result of these scores being overpowered by the contributions of the large individual domains compared to the much smaller domain interfaces.

All six teams mentioned above accurately predicted the structure of the individual HucS_2_L_2_ lobes, with all‐atom nonhydrogen atom RMSDs ranging from 1.38 to 1.80 Å (comparing ~75% of model atoms) between assessed models and the experimental structure (Figure 9C). While the overall architecture of the HucM tetramer and the placement of the HucS_2_L_2_ lobes by all the above‐mentioned groups was approximately correct (Figure 9D), the prediction of interactions between the HucS_2_L_2_ lobes and the HucM scaffold was suboptimal compared to the experimental structure for all models analyzed. In Model 1 by team Yang (H1114TS439_1), which was the highest‐ranked model by lDDT and TM‐score, not all contacts between the HucS_2_L_2_ lobes and the HucM were predicted, compared to the experimental structure, and some clashes were present (Figure 9E–H). This was also true for top models submitted by the teams Zheng (H1114TS374_4), Kiharalab (H1114TS119_1), and McGuffin (H1114TS180_1), with significant clashes and some structural distortion observed. In the top models submitted by Venclovas (H1114TS494_1) and Manifold (H1114TS248_3), the HucS_2_L_2_ lobes were only partially associated with the HucM scaffold.

In conclusion, a number of teams did an impressive job of predicting the architecture of the large multi‐subunit Huc complex. Given the size and complexity of this structure, this is a significant achievement and represents a milestone in computational structural biology. However, no group succeeded in predicting the fine detail of interaction between all subunits of the complex, which significantly impairs further biological interpretation. This highlights the importance of experimental structure determination and indicates that there is still room for improvement in computational methods.

### 2.10 The cryo‐EM structure of EDEM:PDI, the ERAD misfolded glycoprotein checkpoint (CASP: H1157, PDB: 8PKO, EM‐D17749): Provided by Charlie J. Hitchman, Andrea Lia, Yusupha Bayo, and Pietro Roversi

Degradation of terminally misfolded glycoproteins in the endoplasmic reticulum (ER) of eukaryotes is initiated by the checkpoint enzyme of endoplasmic reticulum‐associated degradation (ERAD), a heterodimer formed by a ER degradation‐enhancing α‐mannosidase (EDEM) and its partner protein, a disulfide isomerase (PDI).^88^, ^89^, ^90^, ^91^

The molecular mechanisms underpinning the activity of the EDEM:PDI checkpoint remain unknown. No EDEM nor EDEM:PDI structure has been determined yet. We have determined the 2.7 Å Cryo‐EM structure of the *Chaetomium thermophilum* (*Ct*) EDEM:PDI complex, *Ct*EDEM:*Ct*PDI. The EDEM GH47 catalytic domain nestles inside the curved arc formed by the four thioredoxin domains of the PDI.^92^ Two topologically intertwined C‐terminal *Ct*EDEM domains^93^ stick out of the main body of the complex: the intermediate domain (IMD) is encoded by two nonconsecutive stretches of sequence (*Ct*EDEM 725–820 and 1066–1084) on either side of the protease associated domain (PAD).

Many EDEM sequences show the conservation of two cysteines located in a stretch of sequence that follows the EDEM catalytic GH47 family domain,^94^ At least one of these Cys residues has been putatively implicated in an intermolecular disulfide bond with its partner PDI, based on biochemical data. The disulfide bond is predicted between a free Cys on the mannosidase to the first Cys of one of the PDI redox‐active CXXC motifs.^89^, ^95^ The Cryo‐EM structure reveals that both of these biochemically plausible intermolecular disulfide bridges are actually formed. In the *Ct*EDEM:*Ct*PDI Cryo‐EM structure these two intermolecular disulfides are ^*Ct*PDI^Cys385:^*Ct*EDEM^Cys647 (“SS1”) and ^*Ct*PDI^Cys50:^*Ct*EDEM^Cys719 (“SS2”), providing further evidence that redox chemistry is important for the function of the enzyme.

**FIGURE 10:**
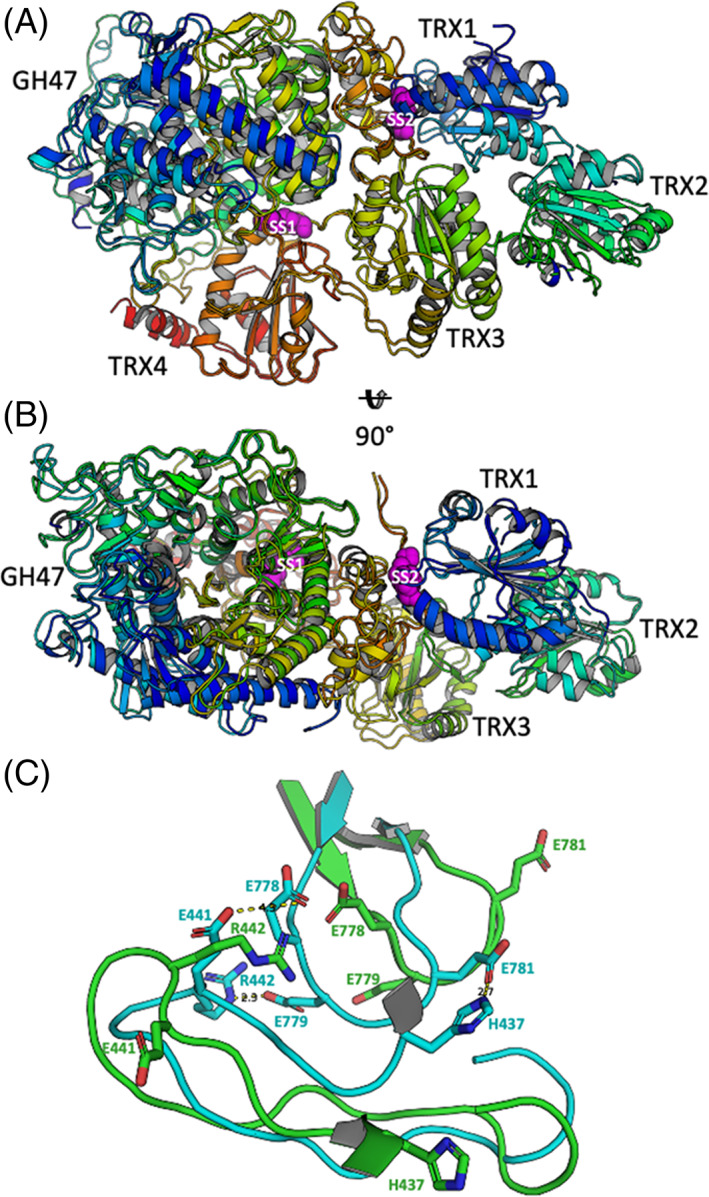
(A and B) *Ct*EDEM GH47 domain and *Ct*PDI. Two views (related by a rotation of 90 degrees around the horizontal axis) of the superposition of the 2.7 Å cryo‐EM structure and the closest CASP15 model (RMSD_Cα_ = 2.7 Å over 1122 residues). The *Ct*EDEM GH47 domain and *Ct*PDI are in cartoon representation and colored from blue to red from N‐ to C‐terminus. The two interchain disulfide bridges are in magenta spheres. (C) The *Ct*EDEM IMD:GH47 interface: overlay of the 2.7 Å cryo‐EM structure (cyan C atoms) with the closest CASP15 model (green C atoms, RMSD_Cα_ = 4.4 Å over 33 residues). IMD residues 774–786 (top) and GH47 residues 436–455 (bottom) in cartoon representation. Three pairs of residues interacting across the interface in the experimental structure (but not in the model) are shown in stick representation, with the distances between their side chains marked by dotted lines: E778:E441, E779:R442, and E781:H437.

Excitingly, 172 of the 208 unique CASP15 predictions manage to place the Sγ of *Ct*PDI Cys50 within 5.0 Å of the Sγ of *Ct*EDEM Cys719, while at the same time placing the Sγ of *Ct*PDI Cys385 within 4.6 Å from the Sγ of *Ct*EDEM Cys647. The top 118 of these structures (57%), which predict an Sγ–Sγ distance in the range 1.8–2.2 Å for both disulfides, also predict a GH47:PDI relative orientation very close to the observed one (see Figure 10A,B), with overall RMSD_Cα_ in the range 2.7–3.4 Å over 1122 residues.

The predictions are worse in the region of the *Ct*EDEM IMD and PAD: this is not entirely surprising given that a number of cryo‐EM 3D classes suggest interdomain mobility. Nevertheless, the main cryo‐EM 3D class allows tracing the IMD:PAD at a local resolution of 3.5–5.0 Å, and the main inter‐domain interface between the GH47 and IMD domains (residues 436–455 and 774–486) is well resolved in the map (cyan C atoms in Figure 10C): yet, none of the CASP15 models correctly predicts the relative orientation of the IMD:PAD tandem domains with respect to the GH47 domain. A few models predict the IMD:PAD intertwined structure reasonably well (RMSD_Cα_ in the range of 3.6–4.0 Å over 369 residues). Perhaps unsurprisingly, the agreement is better for the isolated domains: the best IMD and PAD models have an RMSD_Cα_ around 2.3 Å over 115 and 189 residues, respectively. For this CASP15 target, current protein structure prediction algorithms were better at predicting interactions within the same domain than intramolecular inter‐domain interfaces in this multi‐domain protein.

### 2.11 Structure of the human SUN1‐KASH6 complex (CASP: H1135, PDB: 8B46): Provided by Manickam Gurusaran, Benedikte S. Erlandsen, and Owen R. Davies

The LINC (Linker of Nucleoskeleton and Cytoskeleton) complex spans the nuclear envelope to transduce mechanical forces between cytoskeletal and nuclear components.^96^, ^97^, ^98^ It is formed of nuclear SUN domain proteins and cytoplasmic KASH domain proteins, which interact via their SUN and KASH domains immediately below the outer nuclear membrane.^99^ The LINC complex is essential for nuclear structure and positioning,^96^, ^97^, ^98^ and distinct SUN and KASH protein isoforms have specialized roles, including in sound perception, meiotic chromosome positioning, and gametogenesis.^100^, ^101^, ^102^ Dysfunction of the LINC complex has been associated with various diseases, including muscular dystrophies, neuropathies, and infertility.^98^, ^103^

**FIGURE 11:**
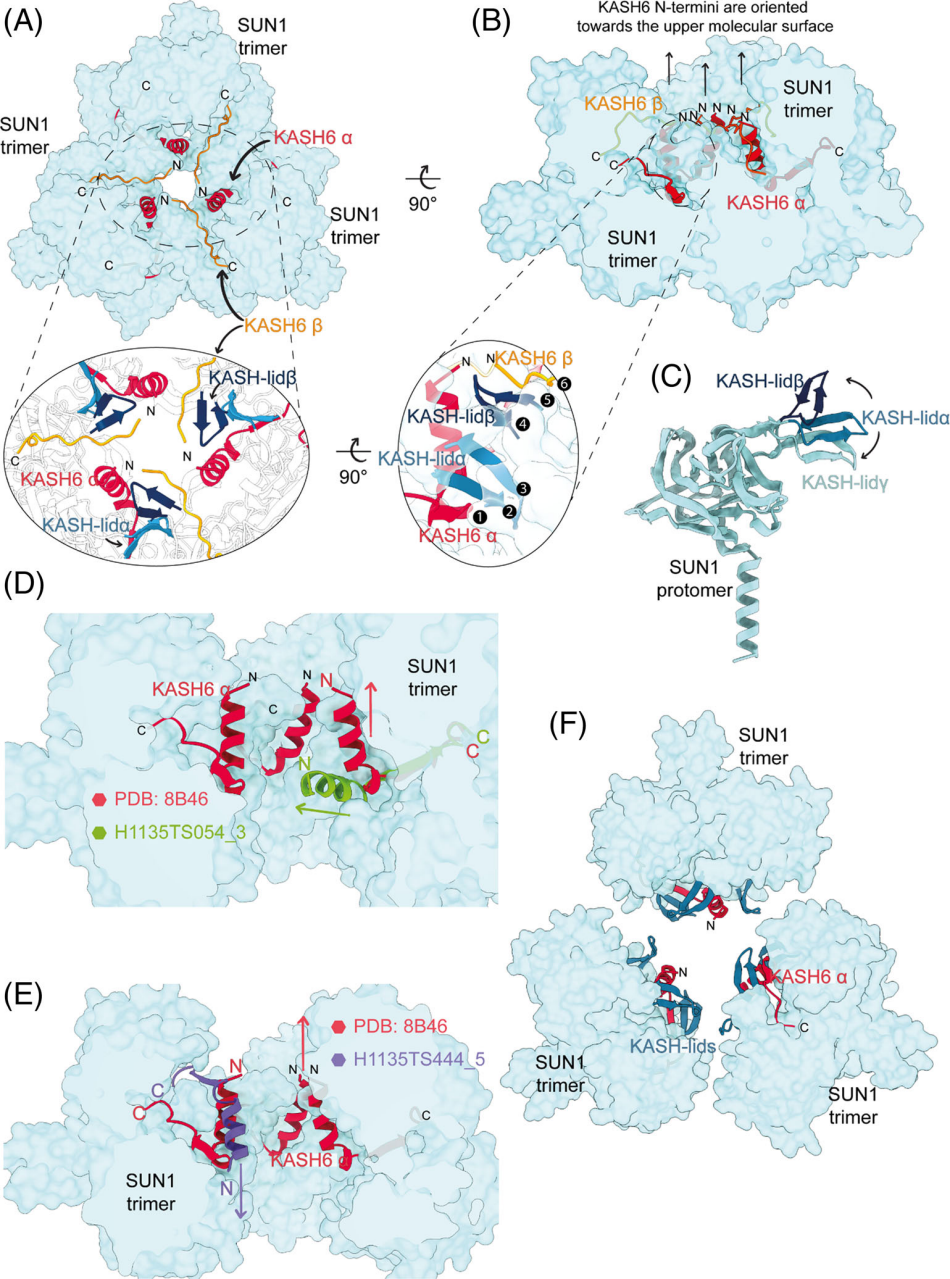
(A,B) Crystal structure of SUN1‐KASH6 exhibiting a “trimer‐of‐trimers” arrangement. (A) KASH6 peptides are represented as cartoons and SUN domains as surfaces and white cartoons in the zoomed panel. (B) The N‐termini of KASH peptides are oriented towards the upper portion of the molecular surface, and at the interface, the KASH peptides together with their KASH‐lids form an extended β‐sheet structure (1–6 in the zoomed panel). (C) Superposition of the three SUN1 protomers from the same trimer of the structure, highlighting the spatial arrangement of the KASH‐lids. (D) KASH peptide from the model H1135TS054_3 (in green) superposed to the crystal structure (with crystal KASH peptide shown in red) (E) The KASH peptide from the model H1135TS444_5 is shown in purple, indicating its inverted orientation compared to the crystal structure peptide (in red), where the N‐terminus of the modeled KASH peptide is incorrectly facing downwards. (F) Model H1135TS086_1 displays a disconnected trimer‐of‐trimers interface, with one of the KASH6 peptides inverted and SUN1 trimers positioned in proximity but with no interface area.

Crystal structures of core SUN‐KASH complexes have previously revealed that SUN domains form globular trimers, with KASH peptides bound between adjacent protomers, assembled head‐to‐head in 6:6 hetero‐oligomers.^104^, ^105^, ^106^ The crystal structure of SUN1‐KASH6, which includes the atypical KASH domain of JAW1/LRMP,^107^ revealed an unusual stoichiometry of nine SUN domains and six KASH peptides, assembled in a ‘trimer‐of‐trimers’ arrangement around a threefold symmetry axis (Figure 11A). Hence, instead of a single head‐to‐head interface, each SUN1 trimer is tightly bound between two surrounding trimers. Each SUN1 trimer is also bound by two KASH6 peptides (KASH6α and KASH6β), each of which has a distinct conformation. KASH6α peptides are well‐ordered, and hook under SUN domain KASH‐lids to form N‐terminal α‐helices. In contrast, KASH6β peptides are poorly‐ordered, and form only β‐sheet interactions with KASH‐lids (Figure 11A,B). The structure has an inherent asymmetry, as the N‐termini of all KASH6 peptides of the 9:6 complex points toward the top surface of the molecule (Figure 11B,C). This is important as their upstream sequences are transmembrane helices that cross the outer nuclear membrane. Hence, the SUN1‐KASH6 9:6 structure describes an arrangement of SUN trimers and KASH peptides that is, in principle, compatible with its known biological positioning immediately below the outer nuclear membrane.

For CASP15, SUN1‐KASH6 was provided as a 9:3 multimeric complex (nine SUN1 domains and the three well‐ordered KASH6α peptides). Most of the predictions reproduced the SUN trimers and their interaction with the C‐termini of KASH peptides, possibly due to the availability of solved SUN‐KASH complexes.^104^, ^105^, ^106^ However, modeling the trimer‐of‐trimers interface and determining the atypical N‐terminal structure of KASH6 peptides presented a challenge. Out of the 309 predictions, ~10% had incorrect stoichiometry and ~40% had incorrect folds or topology (e.g., SUN1 trimers and KASH6 peptides freely suspended in space, or as linear arrays of SUN domains). The crystal structure reveals a highly robust trimer‐of‐trimers interface with 45 potential hydrogen bonds, 15 potential salt bridges, and an interface area of 1467 Å^2^ but ~45% of predictions had disconnected trimer‐of‐trimers structure, meaning the SUN1 trimers were positioned in proximity but with no or very minimal interface area (Figure 11F). The remaining predictions had the correct stoichiometry and overall topology, but with incorrect orientation of KASH6 peptides relative to the trimer‐of‐trimers structure (Figure 11D,E). Hence, even with correct overall trimer‐of‐trimers arrangements, models failed to predict the novelty of the SUN1‐KASH6 complex assembly, in which KASH6 peptides hook under KASH‐lids to form vertically oriented α‐helices. This is important as none of the models suggested the asymmetrical orientation of KASH6 peptides that explains how the structure may form immediately below the outer nuclear membrane, with upstream KASH transmembrane helices seamlessly inserted into the membrane. In summary, these observations illustrate that modeling atypical multimers remains particularly challenging.

### 2.12 The myelin enzyme CNPase bound to the nanobody 8C (CASP: H1142; PDB: N/A): Provided by Sigurbjörn Markússon, Felipe Opazo, and Petri Kursula

Myelin is a highly differentiated proteolipid domain of the plasma membrane of Schwann cells and oligodendrocytes that wraps around selected axons and enables rapid saltatory conduction of nerve impulses. Deficiencies in the formation or maintenance of the multilayered myelin sheath are causative of neurodegenerative diseases, such as multiple sclerosis and peripheral neuropathies. While the reaction catalyzed by 2′,3′‐cyclic nucleotide 3′‐phosphodiesterase (CNPase), an enzyme abundant in myelin, has been known for 60 years,^108^ and CNPase is a widely used marker for myelinating cells, its physiological relevance remains enigmatic.

The phosphodiesterase domain of CNPase has been structurally studied,^109^ but the polynucleotide kinase (PNK)‐like domain has resisted all attempts of high‐resolution structure determination. Therefore, we developed nanobodies against CNPase, to promote both structural and functional studies as well as super‐resolution fluorescence microscopy. Five nanobodies were co‐crystallized with the phosphodiesterase domain of CNPase, and the structures were provided to CASP15 (targets H1140–H1144). The nanobodies had different epitopes, all within the phosphodiesterase domain.

**FIGURE 12:**
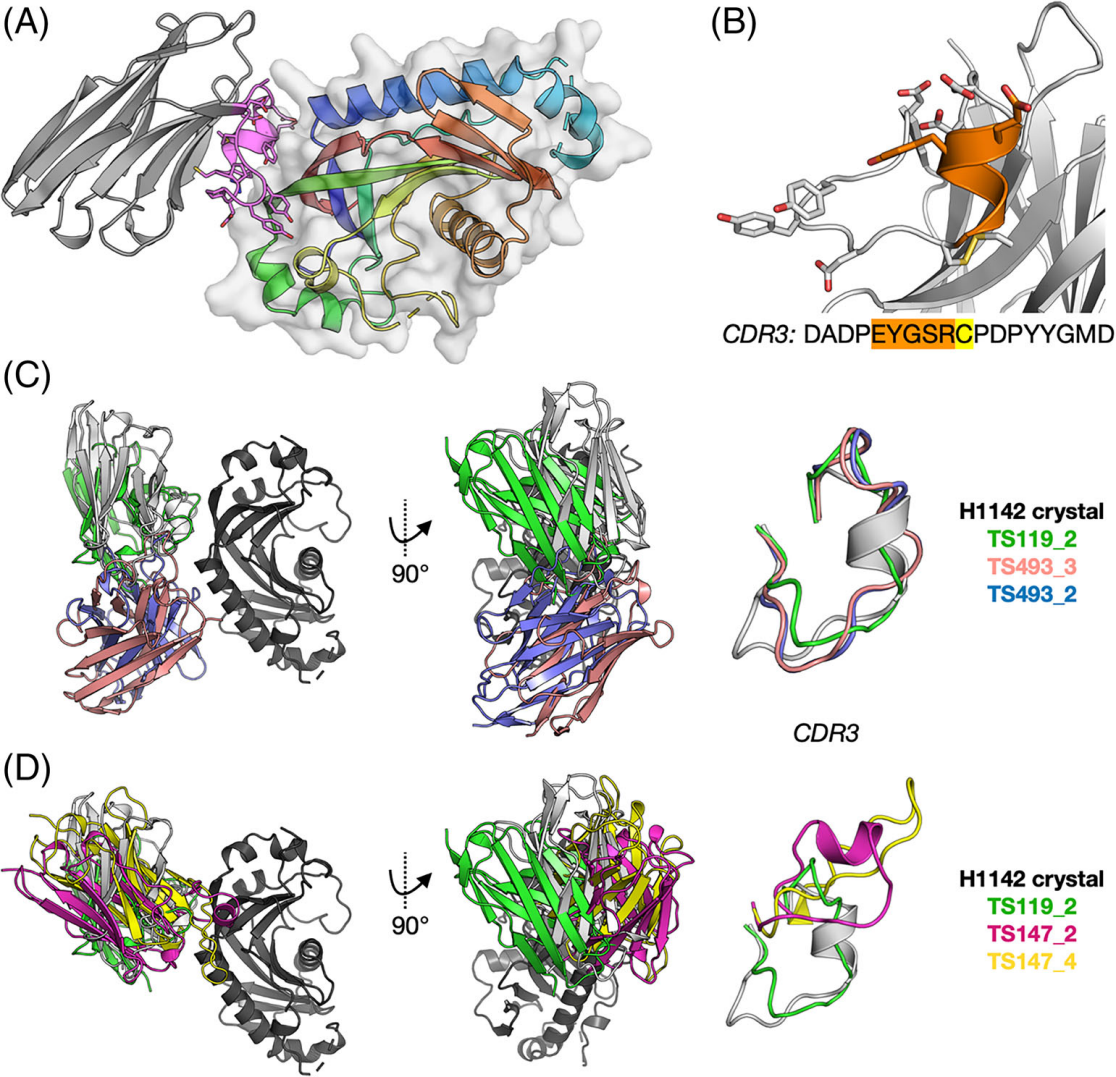
The complex between the CNPase catalytic domain and nanobody 8C. (A) Crystal structure of the complex between the CNPase catalytic domain (surface and rainbow colors) and Nb8C (gray, with the CDR3 loop in purple). (B) Close‐up view of the CDR3 loop, indicating a helical segment held in place by a disulfide bridge. (C) Comparison of the crystal structure to the top three predictions based on the QS score. CNPase is in dark gray and the Nb8C in the crystal structure is in light gray. To the right, a comparison of the CDR3 loop conformation is seen. (D) Comparison of the crystal structure to the top three predictions based on the TMscore. The color coding for (C) and (D) is shown to the right.

The crystal structure (target H1142) of the CNPase catalytic domain with nanobody 8C (Nb8C) revealed Nb8C binding on the “backside” of the domain (Figure 12A). In full‐length CNPase it might also contact the N‐terminal PNK‐like domain. Only the long CDR3 loop of Nb8C (Figure 12A,B) is in contact with CNPase, forming several hydrogen bonds and salt bridges. Three Tyr residues from CDR3 form both regular hydrogen bonds and C–H…π interactions at the interface. The CDR3 loop of Nb8C is bound to the Nb surface via a disulfide bridge, which stabilizes its helical structure.

In CASP15, as the CNPase phosphodiesterase domain structure was known, it was accurately predicted by all groups. For the nanobody, correct prediction would involve both the scaffold and the Nb paratope‐forming loops CDR1–3, which are crucial for epitope recognition. Out of our five submitted CNPase‐nanobody complexes, Nb8C proved to be the most difficult to predict. None of the participants predicted the complex, including the paratope‐epitope interactions, correctly.

We compared the crystal structure to the top three predictions based on the QS (Figure 12C) and TMalign scores (Figure 12D). In both scores, TS119_2 (Kiharalab) was clearly the best prediction, with QS = 0.673, TMscore = 0.773, and interface RMSD = 2.72 Å. These values, however, suggested an at least partially inaccurate prediction of the binding interface even for the highest‐scoring solution. While the approximate binding site in TS119_2 on the CNPase surface is close, the conformation of the nanobody CDR3 loop, and therefore the details of the interaction, are incorrect. The 18‐residue CDR3 loop of Nb8C contains 2 Gly, 3 Pro, and 3 Tyr residues, as well as five acidic residues and one Cys (Figure 12B). While the Tyr, Asp, and Glu are central in CNPase binding, Gly and Pro are likely important for CDR3 conformation, and the Cys covalently links the loop onto the nanobody core.

The comparison highlights the diversity of antibody recognition mechanisms. We believe one reason behind the difficulty of the prediction is the fact that the CDR3 loop of Nb8C involves a disulfide bridge to the nanobody core scaffold, stabilizing the helical segment within the loop. The top prediction TS119_2 brought Nb8C, with a high negative charge on its CDR3 loop, to nearly the correct binding site on the CNPase surface, despite the wrong CDR3 conformation. Future work will involve using the nanobodies as chaperones for solving structures of full‐length CNPase, as well as in functional assays and advanced fluorescence microscopy.

### 2.13 Structure of the nudivirus polyhedrin (CASP: T1122, PDB: 8BBT): Provided by Jeremy R. Keown and Jonathan M. Grimes

Viral occlusion bodies, also known as polyhedra, are native crystals that form an important step in the life cycle of many insect viruses. These occlusion bodies form around the newly assembled virions, with the crystalline occlusion body providing robust protection against many environmental stressors. Occlusion bodies have been observed for double‐stranded DNA viruses (*Baculoviridae*)^110^, ^111^ and double‐stranded RNA viruses (*Reoviridae*).^112^ Although from distant viral lineages, the crystalline lattice formed by these crystals have remarkably similar properties including a conserved cubic unit cell (I23) with unit cell dimensions (101–106 Å, *a* = *b* = *c*). The crystals are built up of a trimeric assembly of the polyhedrin protein with a fold comprising a core of β‐strand strands with α‐helical extensions.^113^

The *Nudiviridae* family of viruses are double‐stranded DNA viruses that share a core set of genes with the *Baculoviridae* and were initially thought not to utilize occlusion bodies as part of their lifecycle.^114^ In 2014, Bézier et al. observed occlusion bodies in a nudivirus that infects the marsh crane fly (*Tipula oleracea*) called *Tipula oleracea* nudivirus.^115^ These viral crystals were first purified in the 1950s, demonstrating their remarkable stability. In this work, we determined the structure of the occlusion body protein that forms these crystals.

These 70‐year‐old crystals were used to determine the polyhedrin structure, revealing a space group (P3_2_21) with unit cell dimensions (*a* = *b* = 53.7 Å, *c* = 105.6 Å), and a dimeric protein building block that is mostly α‐helical. These properties are distinct from those of the previously observed occlusion body proteins. The protein lattice is very dense (solvent content of 22%) and maintained by extensive hydrophobic and electrostatic interactions, disulfide bonds, and domain switching.

**FIGURE 13:**
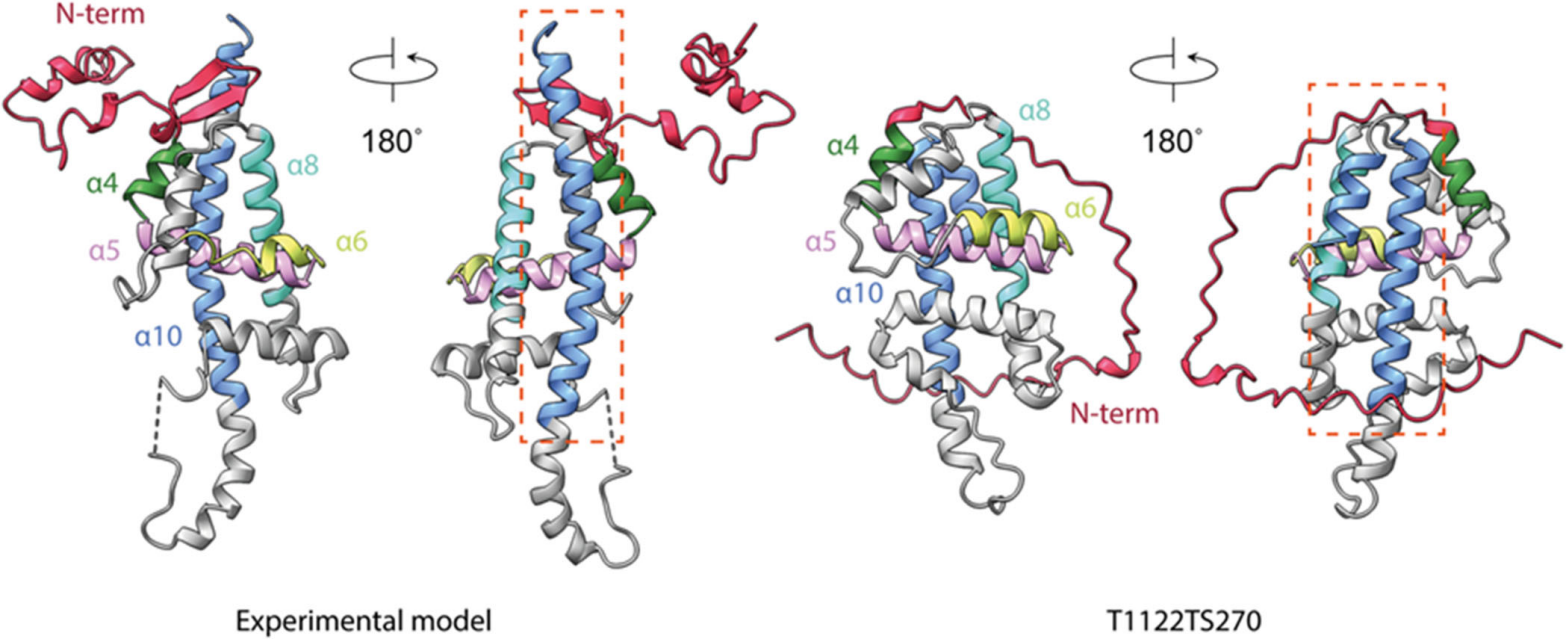
Comparison of the experimental structure and the predicted model T1122TS270_1. Helical regions whose positions were accurately predicted are colored (green, pink, yellow, cyan, and blue). Helix α10 (in blue) was split in the predicted models and is highlighted in the dashed orange box. The N‐terminal region that was not correctly predicted is highlighted in red.

In CASP15, the secondary structure boundaries for the helical core of the protein were predicted correctly by many groups (28 with GDT‐TS 38–40.9) including the top three models of TS257_5, TS427_3, and TS257_4 with GDT‐TS of 40.9, 39.8, and 39.7, respectively. These models were all able to correctly predict the position and relative orientation of helices α4, α5, α6, and sections of α8 and α10 (Figure 13). Only the N‐terminal portion of Helix α8 was correctly positioned, the region close to the core of the protein. Similarly, helix α10 was shortened at the N‐terminal end, compared to the experimental structure, and turns in the opposite direction at residues 224–228 (Figure 13, boxed region). The antiparallel β‐sheet and short helices of the approximately 50 N‐terminal residues in the experimental structure were not predicted accurately by any of the groups. We were unable to use any predicted models for successful molecular replacement. This is due to a mixture of model accuracy and the exceptionally low solvent content of the crystal.

This viral polyhedra is considered to be a difficult target, given the lack of homologous proteins to build a robust multiple sequence alignment, and its numerous stabilizing interactions with neighboring protomers. It is therefore not surprising that the predictors failed to recapitulate many of the features, particularly in the N‐terminal region.

### 2.14 Structure of a *C*. *difficile* extracellular protein of unknown function (CASP: T1176, PDB: 8SMQ): Provided by Monica Rosas‐Lemus, George Minasov, Karla Satchell, and Peter L. Freddolino

*Clostriudium difficile* is an important human pathogen that can cause severe diarrhea and colitis, especially in patients who are immunocompromised and/or have recently been treated with antibiotics.^116^ In the United States alone, there are more than 450 000 cases of *C*. *difficile* infection per year,^117^ resulting in more than 15 000 deaths per year and nearly $5 billion per year in inpatient care costs.^118^ Unfortunately, like many pathogens, the *C*. *difficile* contains many poorly annotated genes, hampering attempts to identify new therapeutic targets. In an effort to identify potential drug or vaccine targets, we recently embarked on a campaign to identify *C*. *difficile* proteins that were expected to reside on the cell surface, highly conserved, and associated with clinical outcomes in large‐scale sequencing of patient‐derived isolates. We initially performed structure and function predictions on a large panel of *C*. *difficile* proteins meeting the criteria listed above, using C‐I‐TASSER^119^ and COFACTOR/MetaGO,^120^, ^121^ respectively. Strikingly, we were able to identify an important subpopulation of *C*. *difficile* for which no confident structural predictions could be obtained, prompting us to propose those proteins as targets for experimental structure determination. Of the difficult‐to‐predict proteins in our *C*. *difficile* pool, CD630_25440 stood out due to a lack of any existing functional annotation, and a low estimated TM‐score of 0.30 for our C‐I‐TASSER predictions.

**FIGURE 14:**
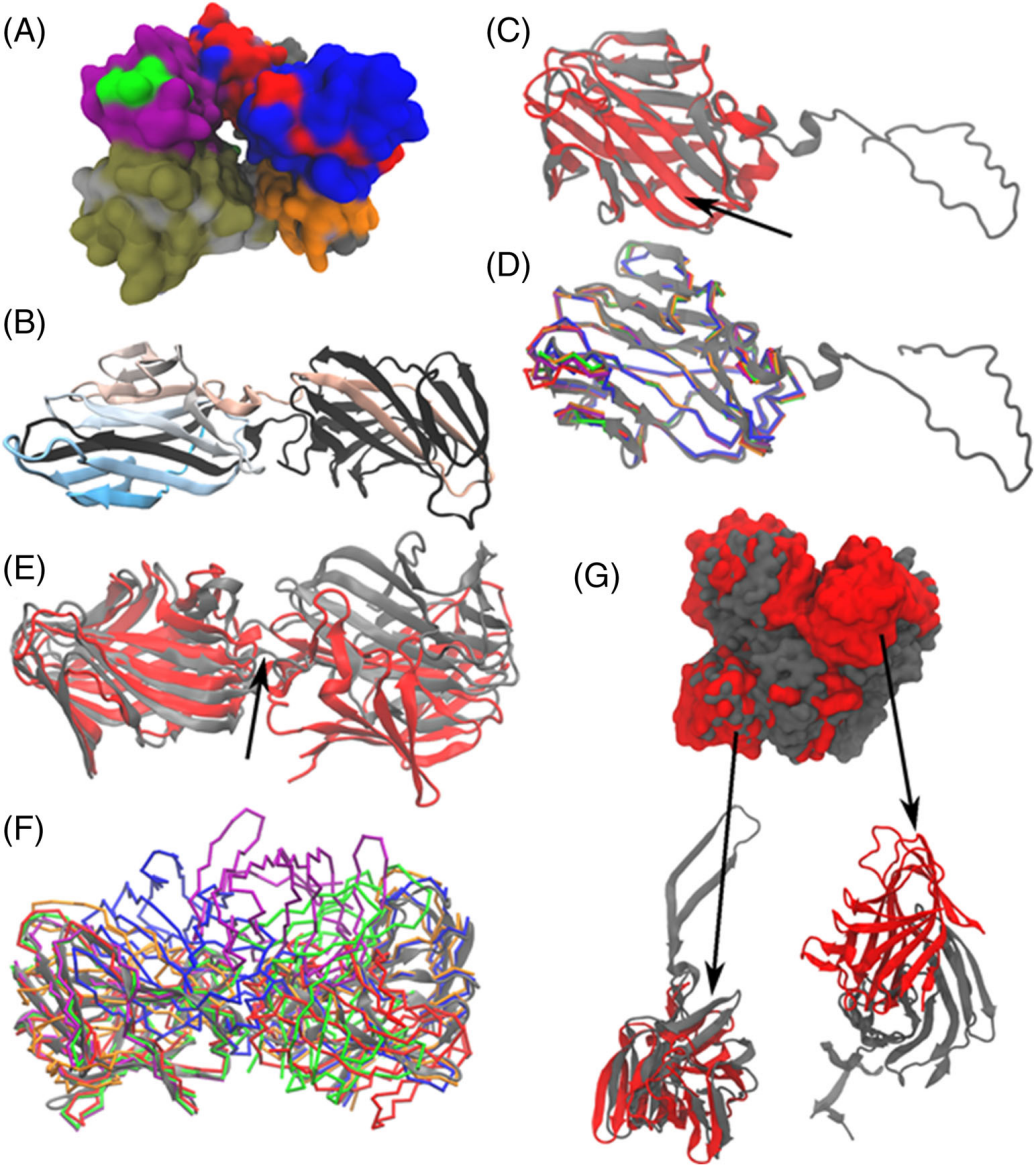
Crystal structure versus CASP15 predictions for target T1176. (A) Overview of the octameric structure of T1176 (CD630_25440). Each chain is shown in a different color. (B) Crystal structure of the T1176 dimer (extracted from the octamer structure), one monomer is shown in black, and the second is colored from blue at the N‐terminus to beige at the C‐terminus. (C) Superposition of the best CASP15 prediction for the T1176 monomer structure (red) versus the crystal structure (gray). The location of a strand that should be flipped out into an adjacent monomer is highlighted with an arrow. (D) As in panel C, with the top five nonredundant CASP15 structure predictions shown in colors ranging from red to purple (in descending order by TM score). (E) Superposition of the best CASP15 structure prediction for the T1176 dimer (red), compared with the crystal structure (gray). The location of an inter‐monomer strand transfer is indicated by the arrow, and is present in the crystal structure but not the predicted structure. (F) As in panel E, showing the top five nonredundant CASP15 predictions. (G) Overview of the top structure prediction for the T1176 octamer (red), versus the crystal structure (gray). Two representative monomers are shown below; one (on the left) with a fairly good superposition in the overall aligned octamer structure, and one (on the right) where the predicted location in the predicted structure differs substantially from the crystal structure.

Based on our analysis of the 358 residue protein, we identified a segment spanning residues 32–202 that were likely suitable for crystallization and capable of forming a single domain structure. Upon experimental structure determination by x‐ray crystallography, we found that CD630_25440 in fact appears to form an octameric structure (Figure 14A), consisting of four dimers. Furthermore, the dimer structure represents a highly unusual fold, with each monomer containing a β‐barrel, but with a pair of β‐strands exchanged between the monomers of each dimer (highlighted in Figure 14B). CD630_25440 was thus provided as a CASP15 target in its monomeric, dimeric, and octameric states.

In contrast to the monomeric unit in the crystal structure, CASP15 predictions uniformly had an intact β‐sheet without the flipped‐out strand. The top group(“bench”, TM‐score of 0.956) predicted a mostly correct fold but incorrectly included the C‐terminal region in the β‐sheet (Figure 14C). Similar trends occur across other top‐performing monomer predictions (Figure 14D).

Predicting multimeric formations of this target proved to be even more difficult. For the CD630_25440 dimeric structure, the highest TM‐score achieved by any group was 0.483 (for the “Manifold‐E” group), which shows both a lack of strand exchange and an incorrect orientation of the monomers relative to each other (Figure 14E). The top five predicted structures had a variety of incorrect dimer conformations (Figure 14F). Similar difficulties persisted in the octameric structure, where the top achieved TM‐score (from the “PEZYFoldings” group) was 0.417, and all structures showed substantial deviations from the correct octameric arrangement (Figure 14G). The unusual β‐strand exchange in CD630_25440 appears to make it an exceptionally challenging target for modern structure prediction methods, possibly due to the difficulty in resolving inter‐ versus intra‐monomer contacts in MSA‐derived contact information.

### 2.15 Mosquito SGS1: salivary gland surface protein 1 from *Aedes aegypti* (CASP: T1169, PDB: 8FJP): Provided by Shiheng Liu, Xian Xia, and Z. Hong Zhou

Pathogen transmission occurs through the process of hematophagy, wherein an infected female mosquito injects its saliva along with potential disease‐causing agents, into a vertebrate host.^122^ Component analyses of mosquito saliva have shown that salivary molecules have anti‐hemostatic and immuno‐modulatory properties which aid blood feeding. Saliva and salivary gland proteins have also been indicated to enhance the severity of transmitted diseases.^123^, ^124^ Among the estimated 100–200 proteins in mosquito saliva, 30%–40% belong to previously uncharacterized protein families with unknown functions.^125^

One of the most abundant salivary proteins in *Aedes aegypti* mosquitoes is a high molecular weight (>300 kDa) protein called salivary gland surface protein 1 (SGS1).^126^ SGS1 is exclusively expressed in the salivary glands of adult female mosquitoes, suggesting its role in blood‐feeding and pathogen transmission.^127^ Screening of monoclonal antibodies enriched for recognition of salivary gland surface epitopes revealed that SGS1 is required for invasion of *Aedes aegypti* salivary glands by *Plasmodium gallinaceum* sporozoites.^127^, ^128^ Reverse genetic studies further confirmed the role of SGS1 in facilitating sporozoite invasion.^129^ Zika virus transmission was also positively affected by SGS1, likely via a similar mechanism.^130^ SGS1 orthologs, including a ~200 kDa protein with neutrophil chemotactic activity from *Anopheles stephensi* saliva^131^ and a ~387 kDa protein with immunomodulatory properties from *Aedes aegypti* saliva,^132^ are thought to promote pathogenicity of arboviruses and *Plasmodium* parasites by modulating the host's immune response.^126^

**FIGURE 15:**
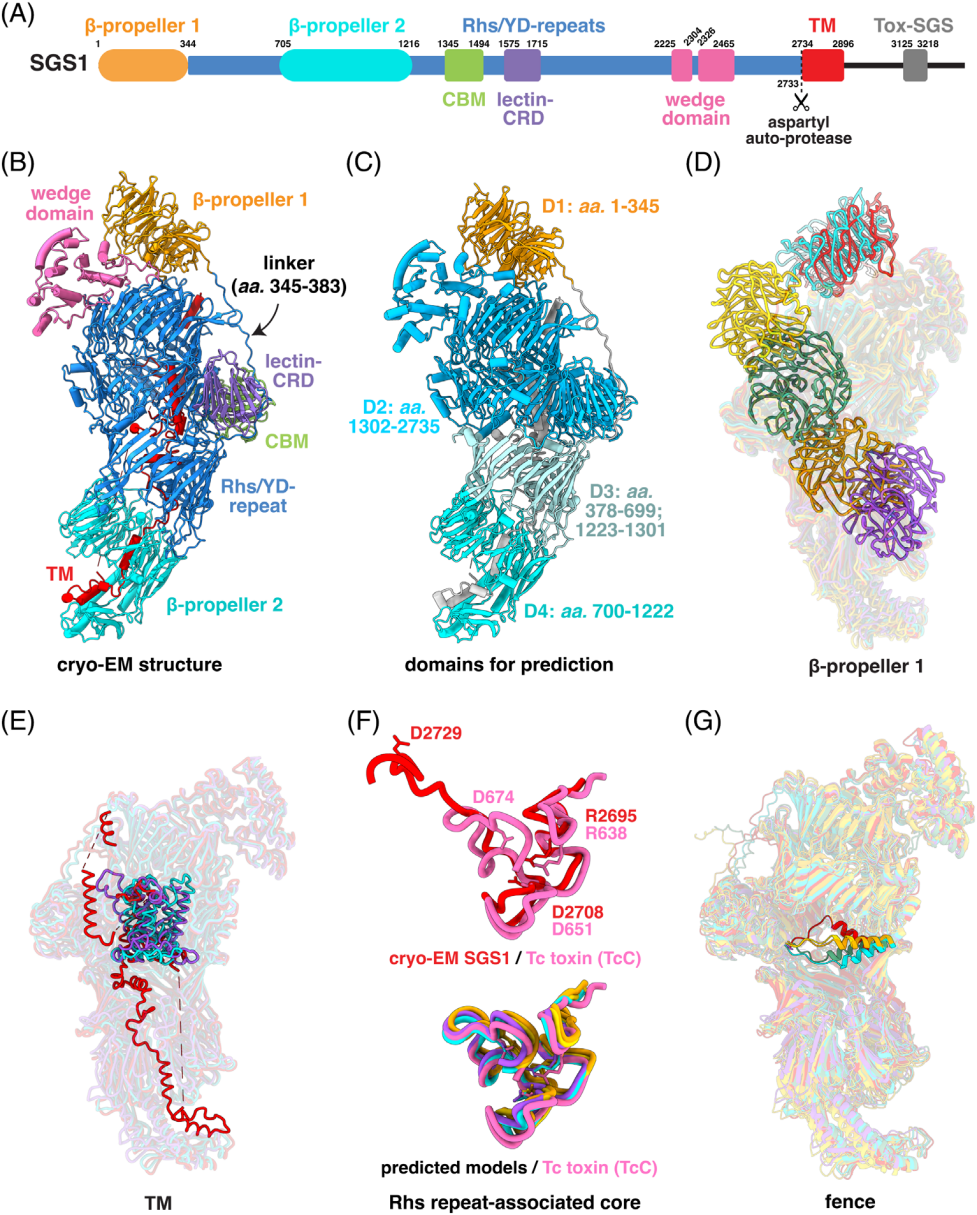
Comparison of CASP15 predictions of mosquito SGS1 with the experimental structure. (A) SGS1 domain diagram. Residue numbers at the domain boundaries are indicated. The putative aspartyl protease cleavage site is shown as dashed line with scissors. β‐propeller 1 (orange), β‐propeller 2 (cyan), Rhs/YD‐repeats (dodger blue), CBM (carbohydrate‐binding module, lime green), lectin‐CRD (lectin carbohydrate‐recognition domain, purple), wedge domain (hot pink), TM (putative transmembrane helices, red), and Tox‐SGS (salivary gland secreted protein domain toxin, gray). (B) Atomic model of SGS1 derived from cryo‐EM, shown in cartoon representation and colored as in A. (C) CASP15 domain segmentation of SGS1 into D1 (orange), D2 (deep sky blue), D3 (pale turquoise), and D4 (cyan). (D–G) Structural comparison of different domains of the cryo‐EM structure of SGS1 with the predicted models: (D) β‐propeller 1; (E) transmembrane; (F) putative aspartyl protease site; and (G) the “fence” that bisects the opening of the daisy‐chained helices. For better visualization, the C‐terminal residues of SGS1 after the aspartyl protease cleavage site and β‐propeller 1 were omitted in (D) and (E), respectively; both were omitted in (G). Color scheme in (D‐G): experimental structures of SGS1 (red) and Tc toxin (pink); predicted structures T1169TS229_1 (cyan), T1169TS278_1 (gold), T1169TS204_1 (orange), T1169TS494_1 (purple), T1169TS074_1 (green).

We recently determined the native structure of SGS1 from mosquito salivary gland by cryo‐EM,^133^ with the *cryoID* approach.^134^ The cocoon‐shaped SGS1 structure is organized into 6 domains: two β‐propeller domains, a rearrangement hotspot/tyrosine‐aspartate (Rhs/YD)‐repeats domain, a carbohydrate‐binding module (CBM), a lectin carbohydrate‐recognition domain (lectin‐CRD), and a wedge domain (Figure 15A,B). The C‐terminal moiety, a ~230 aa‐long sequence previously predicted to form a set of transmembrane helices, (Uniprot ID: Q4VQB1), was surprisingly discovered to be partially folded and almost fully buried within the chamber inside the cocoon shell (red in Figure 15B), explaining how SGS proteins exist in soluble environments. A combination of structural comparison with phylogenetic and sequence analyses uncovered a previously unidentified cleavage site of an aspartic protease, which reconciles the large body of existing biochemical data and suggests a mechanism for transforming and releasing the C‐terminal transmembrane helix‐forming moiety. These helices and numerous receptor domains resolved in the structure likely facilitate sporozoite/arbovirus invasion into the salivary gland or manipulate the host's immune response.

Notably, with its 3364 residues folded into multiple domains with comprehensive domain interactions, SGS1 is the largest monomeric target in the CASP history, and thus serves as a good test for the predictive power of methods in the post‐AlphaFold era. Although it does not have detectable sequence similarity to reported structures, the individual receptor domains—β‐propeller 1 (T1169‐D1), β‐propeller 2 (T1169‐D4), CBM, and lectin‐CRD (T1169‐D2) were well predicted, with the best GDT‐TS ranging from 73 for β‐propeller 1 to 86 for β‐propeller 2, and LDDT ranging from 0.65 for β‐propeller 1 to 0.78 for β‐propeller 2. The interaction of β‐propeller 2, CBM, and lectin‐CRD with the Rhs/YD cocoon shell was also successfully predicted; but the interaction between β‐propeller 1 and the shell was not correctly predicted. The best prediction (Yang group) had a reasonable QS‐score of 0.360 (T1169‐D12: T1169TS229_1; Figure 15D), and poor F1 score (31.6) and Jaccard coefficient (0.30), indicating that only about 30% of the interface contacts agree with the cryo‐EM structure. The successes in predicting inter‐domain interactions are likely due to the facts that CBM and lectin‐CRD are connected to the SGS1 Rhs/YD shell with short linkers and that β‐propeller 2 attaches to the Rhs/YD shell in a similar way as Tc‐toxin. Vice versa, the unsatisfactory performance in predicting the interactions between β‐propeller 1 and the shell is likely due to the fact that β‐propeller 1 is linked to the shell via a long flexible linker (residues 345–383) and no such interface has been identified before.

The central question emerging from our study concerns the potential transformation of the daisy‐chained helices inside the Rhs/YD shell. Remarkably, three important points can be drawn from the incorrectly predicted structures concerning this question. First, no group was able to correctly predict the daisy‐chained helices inside the Rhs/YD shell as shown in the cryo‐EM structure of SGS1. Interestingly, the daisy‐chained helices were incorrectly predicted to be a membrane protein‐like domain inside the Rhs/YD shell (Figure 15E). Should such a predicted structure represent a stable conformation of the daisy‐chained helices after transformation, it would only occur after being released from the Rhs/YD shell and be located outside the cocoon shell in order to access membrane. Second, the cleavage site of aspartic protease in SGS1 was predominantly predicted to exhibit a conformation similar to that of Tc‐toxin (Figure 15F), underscoring the reliance of current prediction algorithms on existing structures in the Protein Data Bank (PDB) for training. Third, the “fence” sequence (residues 1300–1321) that bisects the cocoon opening leading to the daisy‐chained helices was predicted to have various conformations among different modelers (Figure 15G), suggesting a propensity for structural rearrangement near the middle opening of Rhs/YD shell (such as movement of the “fence”) that might serve as a conduit to release these daisy‐chained helices.

### 2.16 Modeling type III secretion proteins YscX:YscY onto the YscV nonamer (CASP: T1106s1, T1106s2, H1106, and H1111, PDB: 7QIJ and 7QIH): Provided by Dominic Gilzer and Hartmut H. Niemann

The target H1111 is a ~590 kDa complex from the *Yersinia enterocolitica* type III secretion system with (approximate) *C*_9_ symmetry. The largest component is the cytosolic domain of the major export gate protein YscV (~40 kDa). YscV is an integral membrane protein. While the structure of the transmembrane domain is unknown, a cryo‐EM structure of the nonameric ring formed by the cytosolic domain is available as template (PDB: 7ALW).^135^ A high‐affinity complex of two smaller proteins (YscX; YscY; ~10 kDa each) binds to the YscV ring with 9:9:9 stoichiometry. While the 7ALW structure follows strict *C*_9_ symmetry, our target structure, determined by x‐ray crystallography, has 18 slightly different copies of the 1:1:1 YscV:YscX:YscY complex in the asymmetric unit.^136^ We provided subsets of this complex as two monomeric and two heteromeric targets. The YscX and YscY protomers were designated as targets T1106s1 and T1106s2, the YscX:YscY heterodimer as target H1106, and the 9:9:9 YscV:YscX:YscY complex as target H1111. The YscX:YscY complex was ranked as an easy target and many predictions matched the published structure.

Our structure of the 9:9:9 YscV:YscX:YscY complex showed that upon binding of YscX:YscY to YscV, there are no major conformational changes in either the YscV ring or the YscX:YscY heterodimer.^136^ Therefore, we had expected modeling the interface between YscV and YscX:YscY to be the main challenge of target H1111. The organizers formulated the task for H1111 on the CASP15 Message Board “… YscX and YscY, had been released as targets T1106s1 and T1106s2 forming H1106, and the third, YscV, is a domain with known structure (PDB: 7ALW). The challenge here is to model the 9:9:9 complex of YscX:YscY:YscV.” However, this task was interpreted differently by different predictors, with only some groups modeling the YscV transmembrane domain, and some groups generating only 1:1:1 complexes. Moreover, the target structure itself has a low resolution (4.1 Å). All these factors led to assessment challenges. Target H1111 has many interfaces including two interactions between YscX and YscY (CASP15:H1106), an interaction between YscV protomers in the ring and several discontinuous interfaces between each YscX:YscY complex and its two adjacent copies of YscV. We as experimentalists were most interested in how well the predictors model the interactions between YscX:YscY and YscV. In contrast, interfaces of the nonameric YscV ring appear to dominate the scoring.

**FIGURE 16:**
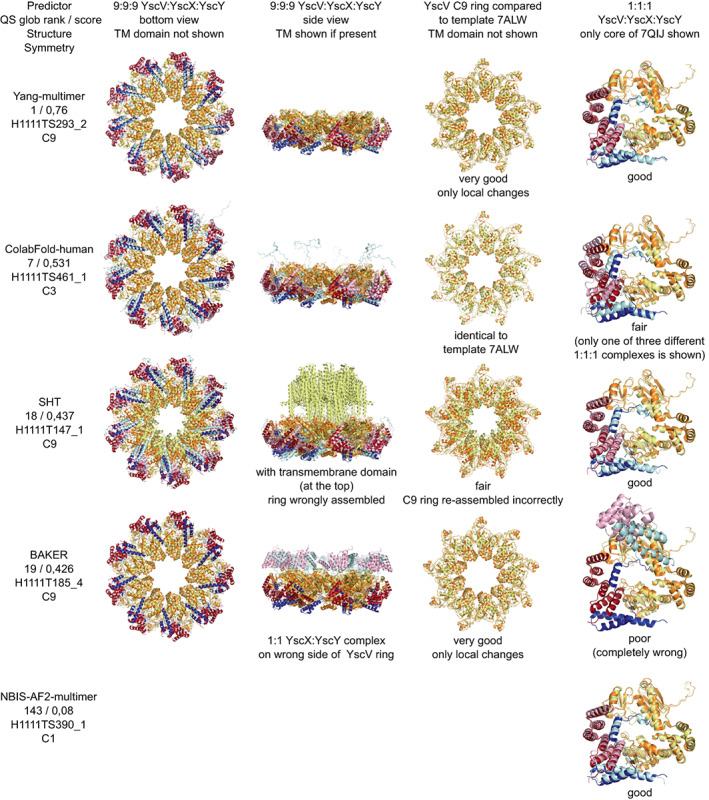
The two left columns show overlays of the predicted 9:9:9 YscV:YscX:YscY complexes (yellow; cyan; pink) on the target structure 7QIJ (orange, blue, and red; chains A*–I*). The third column shows an overlay of the predicted YscV nonamer (yellow) on the YscV template 7ALW (orange). For SHT, the overlay was performed only on a single YscV protomer, shown here at 12 o'clock of the ring. The right column shows an overlay of the predicted 1:1:1 YscV:YscX:YscY complex (yellow; cyan; pink) on one of 18 slightly different 1:1:1 complexes of the target structure 7QIJ (orange; blue; red; chains GA, GB, GC). The structural alignment was performed on YscV only. For the predicted structures, only the region present in 7QIJ is shown.

The Yang‐multimer (ranked 1st) server and some other high‐scoring predictors, for example, Coqualia (ranked 4th), ShanghaiTech (ranked 5th), Yang (ranked 8th), and DFolding‐server (ranked 16th) produced 9:9:9 models with good overall topology and individual interfaces. ColabFold‐human (ranked 7th) left the *C*_9_‐symmetric YscV template unchanged, but modeled a *C*_3_ symmetric 9:9:9 assembly with three different YscV:YscX:YscY complexes, all inferior to that of Yang (ranked 8th). Biologically, this approach does not appear plausible to us. SHT (ranked 18th) and others produced a good 1:1:1 complex but incorrectly re‐assembled the nonameric YscV ring. BAKER was ranked 19th, presumably because they mostly kept the YscV ring with local changes in flexible subdomains, but they wrongly modeled YscX:YscY onto YscV, resulting in a biologically meaningless model. At the same time, the naive 1:1:1 AlphaFold2 model produced a good fit of YscX:YscY to YscV, including the transmembrane domain (Figure 16). Despite its biological relevance, the model received a low score (ranked 143rd), similar to all 1:1:1 models. This suggests that automatic scoring at the complex level may result in misleading top‐ranking models that do not always align with the underlying biology.

Interestingly, some good models contain features that are barely visible in the experimental structure, but may be biologically important. CASP15 models may hence represent a treasure trove for planning of future experiments.

## 3 CONCLUSIONS

This article describes the structural and functional aspects of the selected CASP15 targets. The authors of the structures highlighted the most interesting target features that were reproduced in the models, and also discussed the drawbacks of the predictions.

The overall ability to predict three‐dimensional structures of proteins has remained striking, and many difficult targets were modeled with impressive accuracy. Notably, the most successful prediction methods in both regular and multimeric target categories have leveraged AlphaFold2 as their foundation. These methods include MULTICOM,^137^ MultiFOLD,^138^ Wallner_TS,^139^ Yang‐Multimer, and MEGA‐Protein, all incorporating enhancements in the various steps of the underlying workflow: from improving multiple sequence alignment (MSA) input to rescoring and refining the output models.

The authors asserted that the top models could be used to confidently infer functional sites of the protein. For example, for target T1155, half of the submitted predictions would have led to the same conclusions and prompts for new experiments as derived from the experimental structure. Even for large multi‐protein complexes that are only distantly related to previously described protein structures, as in the case of target H1137, the overall assembly organization could be accurately reproduced. However, for target T1169, the largest monomeric target in the CASP history, prior knowledge such as accurate domain partition and manual intervention (peptide removal), was necessary to enable successful modeling.

Prediction methods struggled when faced with uncommon features that had not been observed in experimental structures. This was evident in cases with the presence of unusual features such as cis‐peptide bonds (T1194), point mutations with substantial effects (T1109 and T1110), atypical stoichiometries (H1135, H1111), and an unexpected topological exchanges (T1176). It is crucial to closely examine these results and related findings to track the ability of advancing methods to accurately reproduce the unconventional structural features that occur in nature.

It is clear that there is room for further improvement, particularly in cases where large conformational flexibility is observed. Specifically, the predictions for the bacteriophage protein (H1129), the nanobody‐bound complex (H1142), and the surface protein 1 (T1169) yielded poor results. Nevertheless, certain alternative conformations, as emphasized by the authors, may represent biologically relevant states and offer valuable insights for a more comprehensive understanding of the structural dynamics of the targets. Likewise, reproducing side‐chain orientations and capturing key interactions, as observed in targets T1194, H1114, H1157, and T1122, remains notably challenging.

The already high accuracy baseline set in CASP14^140^ has been further raised, particularly for multimeric targets. As before, the improvement of methods will continue to heavily rely on the experimental characterization of currently underrepresented structural features and interactions that occur in nature. The current generation of prediction methods continues to serve as an asset for experimentalists when it comes to improving structure determination. In the future, the synergies between computational and experimental methods will be even more instrumental to tackle the existing challenges and identify uncharted areas of the protein universe.

## AUTHOR CONTRIBUTIONS

**Leila T. Alexander, Janani Duraraj, Andriy Kryshtafovych, Krzysztof Fidelis, John Moult, Maya Topf** Conceptualization; writing – review and editing; writing – original draft; coordination. Target‐specific sections: by authors provided in the sections' titles.

4

### PEER REVIEW

The peer review history for this article is available at https://www.webofscience.com/api/gateway/wos/peer‐review/10.1002/prot.26545.

## Supporting information


**TABLE S1.** CASP15 target providers.
**TABLE S2.** The CASP15 target highlights.
