# Supplementary material for: Protein target highlights in CASP15: Analysis of models by structure providers
Source: Proteins. 2023 Jul 26;91(12):1571–99. doi: 10.1002/prot.26545 (PMC10792529; doi:10.1002/prot.26545)
Supplement: Supplementary file 1 — TABLE S1. CASP15 target providers. TABLE S2. The CASP15 target highlights. [file PROT-91-1571-s001.pdf]

SUPPLEMENTARY INFORMATION

Protein target highlights in CASP15: analysis of models by structure providers

Correspondence

Leila Tamara Alexander, email: leila.alexander@unibas.ch

TABLE S1. CASP15 target providers

#	Contributor	Country	Institution	Target
1	Luciano A. Abriata	Switzerland	EPFL	T1155
2	Yusupha Bayo	Italy	U of Milano	T1157
3	Gira Bhabha	USA	New York U	H1137
4	Cécile Breyton	France	IBS, Grenoble	H1129
5	Simon G Caulton	UK	U of Birmingham	T1194
6	James Chen	USA	New York U	H1137
7	Séraphine Degroux	France	IBS, Grenoble	H1129
8	Damian Ekiert	USA	New York U	H1137
9	Benedikte S. Erlandsen	UK	U of Edinburgh	H1135
10	Peter L. Freddolino	USA	U of Michigan	T1176
11	Dominic Gilzer	Germany	Bielefeld U	T1106s1, T1106s2, H1106 and H1111
12	Chris Greening	Australia	Monash U	H1114
13	Jonathan M. Grimes	UK	U of Oxford	T1122
14	Rhys Grinter	Australia	Monash U	H1114
15	Manickam Gurusaran	UK	U of Edinburgh	H1135
16	Marcus D. Hartmann	Germany	Max Planck, Tübingen	T1180
17	Charlie J. Hitchman	UK	U of Leicester	H1157
18	Jeremy R. Keown	UK	U of Oxford	T1122
19	Ashleigh Kropp	Australia	Monash U	H1114
20	Petri Kursula	Norway	U of Bergen	H1142
21	Andrew L Lovering	UK	U of Birmingham	T1194
22	Bruno Lemaitre	Switzerland	EPFL	T1155
23	Andrea Lia	UK	U of Leicester	H1157
24	Shiheng Liu	USA	U of California	T1169
25	Maria Logotheti	Germany	Max Planck, Tübingen	T1180
26	Shuze Lu	China	Lanzhou U	T1183
27	Sigurbjörn Markússon	Norway	U of Bergen	H1142
28	Mitchell D. Miller	USA	Rice U	T1124 and T1124o
29	George Minasov	USA	Northwestern U	T1176
30	Hartmut H. Niemann	Germany	Bielefeld U	T1106s1, T1106s2, H1106 and H1111
31	Felipe Opazo	Germany	NanoTag Biotechnologies	H1142
32	George N. Phillips, Jr.	USA	Rice U	T1124 and T1124o

33	Owen R. Davies	UK	U of Edinburgh	H1135
34	Samuel Rommelaere	Switzerland	EPFL	T1155
35	Monica Rosas-Lemus	USA	Northwestern U	T1176
36	Pietro Roversi	UK	U of Leicester	H1157
37	Karla Satchell	USA	Northwestern U	T1176
38	Nathan Smith	USA	U of Nebraska	T1109 and T1110
39	Mark A. Wilson	USA	U of Nebraska	T1109 and T1110
40	Kuan-Lin Wu	USA	Rice U	T1124 and T1124o
41	Xian Xia	USA	U of California	T1169
42	Han Xiao	USA	Rice U	T1124 and T1124o
43	Wenhua Zhang	China	Lanzhou U	T1183
44	Z. Hong Zhou	USA	U of California	T1169

TABLE S2. The CASP15 target highlights

Target	PDB	Length, aa	Method	Res, Å	Stoichiom	GDT-TS	lDDT	QS-score	GDT-TS	lDDT	QS-score
T1183	8IEY	200	X-Ray	2.00	A1	97.95	0.91		97.95	0.91	
T1155	N/A	116	NMR	-	A1	72.60	0.62		72.11	0.62	
T1124	7UX8	384	X-Ray	1.20	A2	90.28	0.88		89.55	0.86	
T1124o					A2			0.93			0.93
H1137	8FEF	3939	EM	2.71	A1B1C1D1 E1F1G2H1I1			0.90			0.89
T1180	N/A	404	X-Ray	2.47	A1	88.65	0.88		88.39	0.88	
T1194	8OKH	168	X-Ray	2.15	A1	99.69	0.82		99.53	0.90	
T1109	N/A	227	X-Ray	1.00	A2	94.16	0.90		93.92	0.90	
T1110	N/A	227	X-Ray	0.74	A2	97.62	0.94		97.06	0.93	
H1129	8B14	1387	EM	2.60	A1B1			0.85			0.82
H1114	7UUS, 7UTD, 7UUR, 8DQV	1093	EM	1.52-8.00	A4B8C8			0.85			0.85
H1157	8PKO, EMD-17749	1122	EM	2.70	A1B1			0.80			0.80
H1135	8B46	220	X-Ray	1.70	A9B3			0.72			0.72
H1142	N/A	347	X-Ray	1.73	A1B1			0.67			0.28
T1122	8BBT	241	X-Ray	1.69	A1	39.13	0.50		39.13	0.50	
T1176	8SMQ	170	X-Ray	2.00	A1	92.94	0.81		92.65	0.81	
T1176o		1360			A8			0.07			0.07
T1176v1o		340			A2			0.09			0.09
T1169	8FJP	3364	EM	3.30	A1	57.72	0.63		54.63	0.67	
T1169-D1		345				72.68	0.63		70.51	0.63	
T1169-D2		1434				77.32	0.77		74.02	0.76	
T1169-D3		401				85.85	0.81		84.97	0.78	
T1169-D4		322				85.71	0.78		85.71	0.78	
T1106s1	7QIH	122	X-Ray	1.92	A1	85.92	0.78		85.92	0.78	
T1106s2		114	X-Ray	1.92	A1	95.72	0.90		95.72	0.89	
H1106		236	X-Ray	1.92	A1B1			0.88			0.88
H1111	7QIJ	940	X-Ray	4.10	A9B9C9			0.71			0.69
